# *De novo* Inference of Diversity Genes and Analysis of Non-canonical V(DD)J Recombination in Immunoglobulins

**DOI:** 10.3389/fimmu.2019.00987

**Published:** 2019-05-03

**Authors:** Yana Safonova, Pavel A. Pevzner

**Affiliations:** ^1^Center for Information Theory and Applications, University of California, San Diego, San Diego, CA, United States; ^2^Department of Computer Science and Engineering, University of California, San Diego, San Diego, CA, United States

**Keywords:** repertoire sequencing, VDJ recombination, germline gene inference, antibody repertoire, repertoire diversity

## Abstract

The V(D)J recombination forms the immunoglobulin genes by joining the variable (V), diversity (D), and joining (J) germline genes. Since variations in germline genes have been linked to various diseases, personalized immunogenomics aims at finding alleles of germline genes across various patients. Although recent studies described algorithms for *de novo* inference of V and J genes from immunosequencing data, they stopped short of solving a more difficult problem of reconstructing D genes that form the highly divergent CDR3 regions and provide the most important contribution to the antigen binding. We present the IgScout algorithm for *de novo* D gene reconstruction and apply it to reveal new alleles of human D genes and previously unknown D genes in camel, an important model organism in immunology. We further analyze non-canonical V(DD)J recombination that results in unusually long CDR3s with tandem fused IGHD genes and thus expands the diversity of the antibody repertoires. We demonstrate that tandem CDR3s represent a consistent and functional feature of all analyzed immunosequencing datasets, reveal ultra-long CDR3s, and shed light on the mechanism responsible for their formation.

## Introduction

Antibodies provide specific binding to an enormous range of antigens and represent a key component of the adaptive immune system. The *antibody repertoire* is generated by *somatic recombination* of the V (*variable*), D (*diversity*), and J (*joining*) germline gene segments. Immunosequencing has emerged as a method of choice for generating millions of reads that sample antibody repertoires and provide insights into monitoring immune response to disease and vaccination (1).

Information about all germline genes in an individual is a pre-requisite for analyzing immunogenomics data. However, nearly all immunogenomics studies rely on the population-level germline genes rather than germline genes in a specific individual that the immunosequencing data originated from. This approach is deficient since the set of known germline genes is incomplete (particularly for non-Europeans) and contains alleles that resulted from sequencing and annotation errors (2, 3). Moreover, it is non-trivial to figure out which known allele(s) is present in a specific individual since the widespread practice of aligning each read to its closest germline gene results in high error rates (3). These errors hide the identity of the individual germline genes, make it difficult to analyze *somatic hypermutations* (*SHM*) and complicate studies of antibody evolution (4–6).

*Personalized immunogenomics* (i.e., identifying individual germline genes) is important since variations in germline genes have been linked to various diseases (7), differential response to infection, vaccination, and drugs (8, 9), aging (10), and disease susceptibility (7, 11, 12). However, since the International ImMunoGeneTics (IMGT) database is incomplete even in the case of well-studied human germline genes (13), there exist still unknown human allelic variants that are difficult to differentiate from SHMs. In the case of immunologically important but less studied model organisms, such as camels or sharks, the germline genes remain largely unknown. Unfortunately, since assembling the highly repetitive immunoglobulin locus from whole genome sequencing data faces challenges (14), the efforts like the 1,000 Genomes Project have resulted only in limited progress toward inferring the population-wide census of germline genes (14–16).

In addition to personalized immunogenomics, the incompleteness of the IMGT database negatively affects analysis of monoclonal antibodies. Existing tools for antibody sequencing from tandem mass spectra (17, 18) rely on a comprehensive database of V, D, and J genes to assemble tandem mass spectra into an intact antibody. Lack of such databases for many species limits applications of Valens (Digital Proteomics), SuperNova (Protein Metrics), and other software tools for antibody sequencing.

Although the personalized immunogenomics approach was first proposed by Boyd et al. (19), the manual analysis in this study did not result in a software tool for inferring germline genes. Gadala-Maria et al. (20) developed the TIgGER algorithm for inferring germline genes and used it to discover 11 novel allelic V segments. However, 20 stopped short of *de novo* reconstruction of the germline genes and acknowledged that it is important to develop algorithms for finding diverged alleles that TIgGER is not able to find. In the case of V and J genes, this challenge was addressed by Corcoran et al. (21), Zhang et al. (22), and Ralph and Matsen (3). However, as Ralph and Matsen (3) commented, the more challenging task of *de novo* reconstruction of D genes remains elusive. This is unfortunate since D genes contribute to the *complementarity determining region 3* (*CDR3*) that covers the junctions between V, D, and J genes and represents the highly divergent part of antibodies. We describe the IgScout algorithm for *de novo* inference of D genes and apply it to diverse immunosequencing datasets with the goal to reconstruct dominant variants of highly abundant D genes and discover novel highly abundant variations.

Although many studies analyzed patterns of V-D-J pairing (23, 24), there is still a shortage of studies of unusual recombination events such as *V(DD)J recombination* incorporating two D genes into a single unusually long CDR3 with tandem fused IGHD genes (or *tandem CDR3*). Meek et al. (25) were the first to reveal a few tandem CDR3s, thus confirming the V(DD)J recombination conjecture put forward by Kurosawa and Tonegawa (26). However, since tandem CDR3s are rare, they remained elusive for the next two decades and (27, 28) even argued that tandem CDR3s found in Meek et al. (25) represent artifacts. However, Briney et al. (29) and Larimore et al. (30) demonstrated that tandem CDR3s do exist (at frequency 1 per 800 B-cells) by analyzing high-throughput immunosequencing datasets.

As emphasized in Briney et al. (29), detecting V(DD)J recombination has to be done with caution since it is often confused with standard V(D)J recombination. Although they came up with a heuristic for detecting tandem CDR3s, there is still no software for detecting tandem CDR3s and it remains unclear how many tandem CDR3s found in Briney et al. (29) represent false positives. We thus extended the functionality of the IgScout algorithm to finding tandem CDR3s and revealed that V(DD)J recombination is a functional (rather than aberrant) feature with frequency varying from 1 per 200 to 1 per 2,500 B-cells across various datasets. Finally, we revealed *ultra-long tandem CDR3s* and shed light on the mechanism responsible for their formation.

## Results

### Immunosequencing Datasets

We analyzed the following datasets described in the Supplemental Note “Immunosequencing datasets”:

- **HEALTHY**: 14 datasets from 14 healthy human donors,
- **ALLERGY**: 24 datasets from six allergy patients (31),
- **HIV**: 13 datasets from two HIV-infected patients (32),
- **NAÏVE:** 7 datasets from naïve B cells of healthy human donors,
- **PROJECT10**: 600 datasets from various humans resulting from 10 NCBI projects
- **CAMEL**: 6 datasets from three healthy camels (33).

### Constructing CDR3 Datasets

We illustrate the work of IgScout using one of the HEALTHY datasets (Set 1) containing heavy chain repertoires extracted from *peripheral blood mononuclear cells* (*PBMC*). The IgReC tool (34) extracted 228,619 distinct CDR3s from this dataset. To minimize impact of sequencing and amplification errors, we clustered similar CDR3s (differing by at most three mismatches) and constructed consensus for each cluster resulting in 98,576 *consensus CDR3* of average length 46 nucleotides.

Each CDR3s typically starts from a short suffix of a V gene and ends with a short prefix of a J gene. Since these suffixes and prefixes negatively affect reconstruction of D genes, IgScout trims them as described in the Supplemental Note “Preprocessing CDR3 datasets.” This procedure reduces the average length of CDR3 strings (46 nucleotides) to 30 nucleotides strings that represent substrings of CDR3s that are not encoded by IGHV or IGHJ genes. The result of the procedure is the set of strings CDR3^*^. We refer to the number of strings in CDR3^*^ as |CDR3^*^|.

### Overview of Human D Genes

The human immunoglobulin (IGH) locus contains 27 D genes that vary in length from 11 to 37 nucleotides. Since two pairs of human D genes are identical, there exist only 25 distinct D genes. Since the IMGT database refers to D genes using rather long names and since these names do not reveal the ordering of D genes in the IGH loci (that is important for analyzing tandem CDR3s), it is difficult to visualize the IgScout results across all D genes and across multiple immunosequencing datasets. We thus renamed distinct human D genes from D1 to D27 in the increasing order of their positions in the IGH locus. The IMGT database also contains seven alleles of D genes denoted D2^*^2, D2^*^3, D3^*^2, D8^*^2, D10^*^2, D16^*^2, and D21^*^2. See Table 1 and Supplemental Note “Information about human D genes” for details.

**Table 1:** Positions and lengths of human D genes.

**Name**	**IMGT name**	**Position (bp)**	**Length (nt)**	**Name**	**IMGT name**	**Position (bp)**	**Length (nt)**
D1	IGHD1-1	105,919,502	17	D15	IGHD2-15	105,897,957	31
D2	IGHD2-2	105,916,826	31	D16	IGHD3-16	105,895,634	37
D3	IGHD3-3	105,914,359	31	D17	IGHD4-17	105,894,508	16
D4	IGHD4-4	105,913,222	16	D5	IGHD5-18	105,893,542	20
D5	IGHD5-5	105,912,257	20	D19	IGHD6-19	105,891,699	21
D6	IGHD6-6	105,910,410	18	D20	IGHD1-20	105,891,191	17
D7	IGHD1-7	105,909,907	17	D21	IGHD2-21	105,888,551	28
D8	IGHD2-8	105,907,211	31	D22	IGHD3-22	105,886,031	31
D9	IGHD3-9	105,904,681	31	D23	IGHD4-23	105,884,870	19
D10	IGHD3-10	105,904,497	31	D24	IGHD5-24	105,883,903	20
D4	IGHD4-11	105,903,616	16	D25	IGHD6-25	105,881,539	18
D12	IGHD5-12	105,902,649	23	D26	IGHD1-26	105,881,034	20
D13	IGHD6-13	105,901,142	21	D27	IGHD7-27	105,865,551	11
D14	IGHD1-14	105,900,638	17				

Since the IGH locus starts at the end of the 14th chromosome, positions are given with respect to its complementary sequence (assembly GRCh38.p12). Green and orange cells correspond to two duplicated and identical D genes IGHD4-4*01–IGHD4-11*01 (D4) and IGHD5-5*01–IGHD5-18*01 (D5).

### Frequent *k*-mers in D Genes

The problem of inferring germline genes can be formulated as the Trace Reconstruction Problem (35) in information theory described in the Methods section. IgScout is a heuristic for solving this problem that is inspired by the RepeatScout algorithm for *de novo* repeat finding (36) and that is based on analyzing frequent *k*-mers (contiguous strings of length *k*) in CDR3s. We illustrate the work of IgScout using *k*-mers of size 15 (all human D genes are longer than 15 nucleotides except for 11 nucleotide long gene D27).

The human D genes contain 305 15-mers. We classify a *k*-mer as *known* if it occurs in a human D gene (from IGHD1-1 to IGHD7-27), *mutated* if it differs from a known *k*-mer by a single substitution, and *trimmed* if it contains a known (*k*-2)-mer. All other *k*-mers are called *foreign*. Twenty-seven percent of strings in the CDR3^*^ dataset contain a known 15-mer and 35% contain either a known, or a mutated, or a trimmed 15-mer.

**Figure 1:**
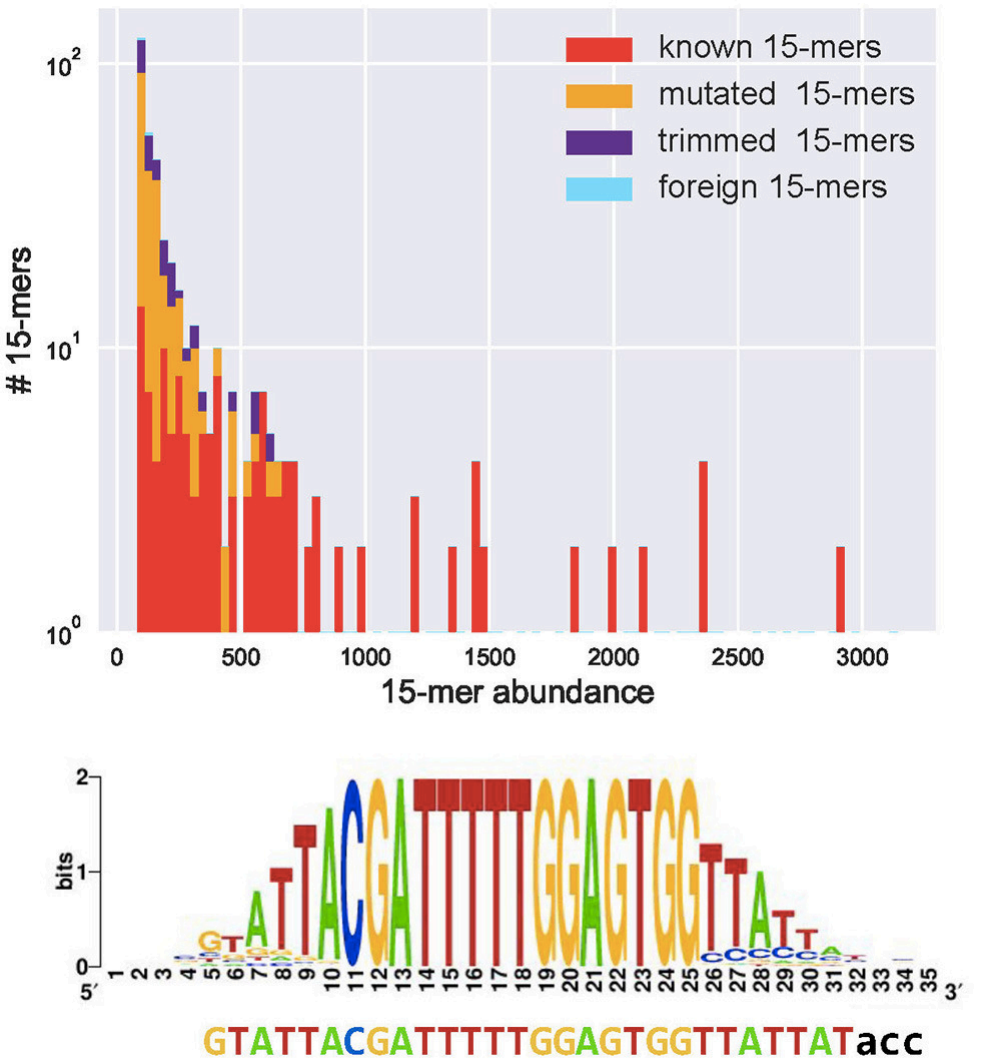
Abundances of all 443 common 15-mers **(top)** and the motif logo constructed for the most abundant 15-mer CGATTTTTGGAGTGG in the CDR3* dataset constructed from the Set 1 dataset **(bottom)**. **(Top)** The CDR3* dataset contains 91% of all 15-mers appearing in human D genes (all 15-mers in human D genes are unique, i.e., appear in a single D gene). Four hundred forty-three common 15-mers in the CDR3* set have abundances varying from 83 to 3,141. The *y*–axis represents the number of common 15-mers with given abundance (in logarithmic scale). Red, yellow, violet, and blue bars represent the number of common 15-mers with given abundance among known, mutated, trimmed, and foreign 15-mers, respectively. There exist 175 known, 195 mutated, 70 trimmed, and three foreign common 15-mers. The histogram represents 100 bins of width 30 each. **(Bottom)** The ATTACGATTTTTGGAGTGGTTAT is the initial 28-nucleotide long sequence formed by positions in the motif logo with high information content (37). The motif logo was constructed using 3,141 sequences from the set CDR3* containing the most abundant *k*-mer. After extending this 28-mer, IgScout reconstructed the 30-mer GTATTACGATTTTTGGAGTGGTTATTAT that is a substring of the 33-nucleotide long IGHD3-3 gene GTATTACGATTTTTGGAGTGGTTATTAT acc shown below the logo.

We classify a *k*-mer as *common* if its abundance exceeds fraction^*^ |CDR3^*^| (the default value *fraction* = 0.001). Figure 1 and the Supplemental Note “Common *k*-mers” present distributions of frequencies of all common 15-mers in various datasets. Although the vast majority of common *k*-mers are known, mutated, or trimmed, some of them are foreign. These foreign common *k*-mers have to be treated with caution since they may trigger false positive inferences of D genes.

### From Frequent *k*-mers to D Gene Reconstruction

**Figure 2:**
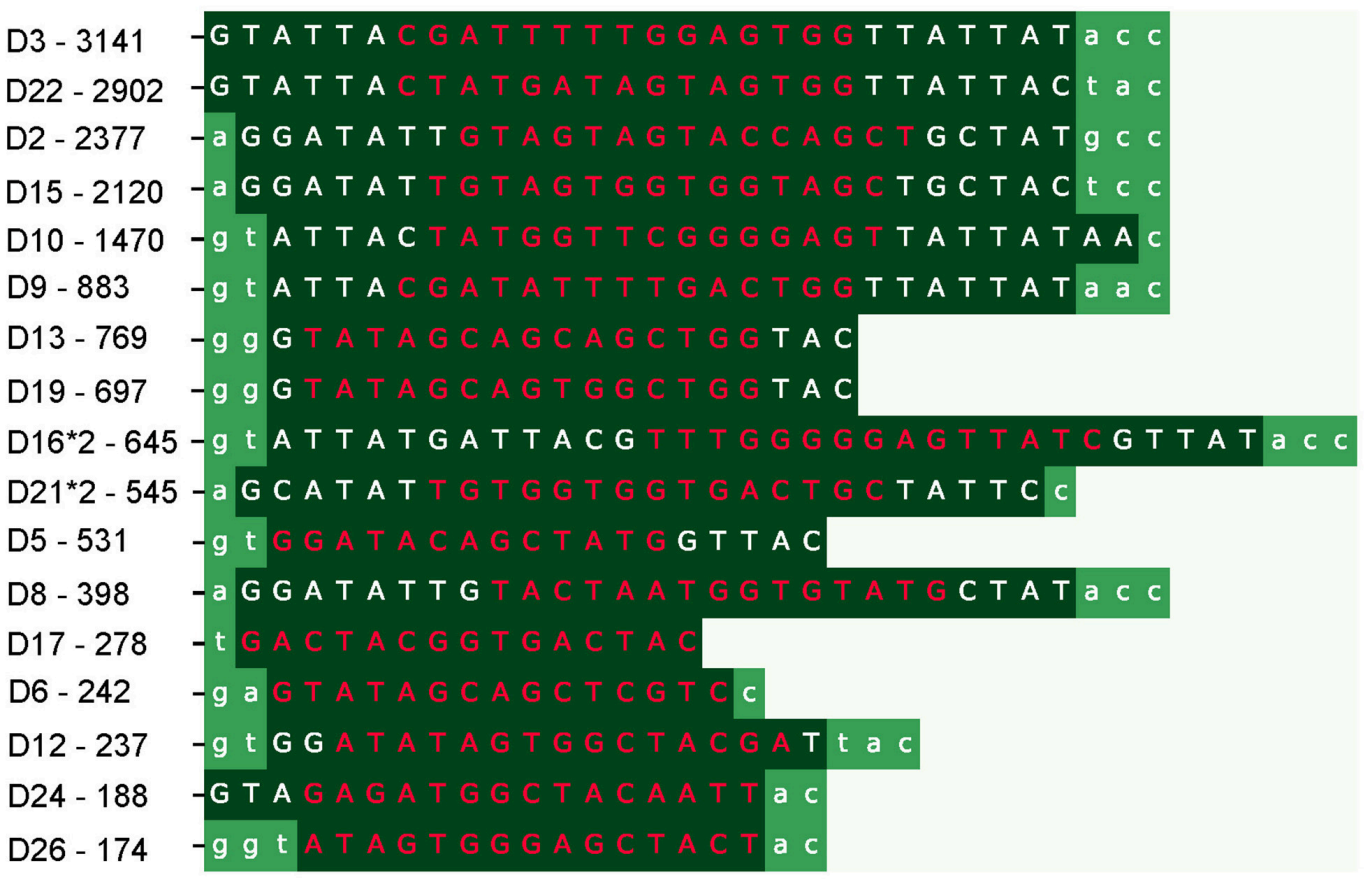
IgScout results on the CDR3* dataset. Each row shows a reconstructed string (strings are inferred in the order from the top to the **bottom**). Dark green segments correspond to reconstructed substrings of human D genes (flanking non-reconstructed nucleotides are shown in standard green). The most frequent 15-mers that were used for reconstructing the corresponding D genes are shown in red (their abundances are shown on the left). The reconstructed substring of the D2 gene (IGHD2-2) also occurs in D2*2 and D2*3 genes. Seventeen strings reconstructed by IgScout represent substrings of 17 human D genes. IgScout misses short prefixes and suffixes of D genes: 1.4 nucleotides on the left and 1.7 nucleotides on the right, on average for the Set 1 dataset (0.9 nucleotides on the left and 1.5 nucleotides on the right, on average after combining reconstructions over all HEALTHY datasets). IgScout did not reconstruct eight human D genes: D1 (IGHD1-1), D4 (IGHD4-4), D7 (IGHD1-7), D14 (IGHD1-14), D20 (IGHD1-20), D23 (IGHD4-23), D25 (IGHD6-25), and D27 (IGHD7-27) that contributed to few CDR3 in the Set 1. These genes have the following abundances of their most frequent 15-mers: 43 for D1, 59 for D4, 83 for D7, 0 for D14, 33 for D20, 75 for D23, 0 for D25, and 0 for D27.

IgScout selects a most abundant *k*-mer in the CDR3^*^ dataset, aligns all CDR3 that contain this *k*-mer (using this *k*-mer as the alignment seed), and constructs the *motif logo* of the resulting alignment (Figure 1). It further trims all positions of the motif logo with the *information content* below *IC* (the default value *IC* = 0.5) and computes the consensus string. Afterwards, it extends the consensus strings to the right and to the left (the PrefixExtension and SuffixExtension steps in the Supplemental Note “IgScout pseudocode”) to construct a putative D gene as described in the Methods section. Finally, the algorithm removes the sequences that contain *k*-mers from the identified putative D gene from the set CDR3^*^, finds a most abundant *k*-mer in the resulting dataset, and iterates. IgScout stops when a most abundant *k*-mer is not a common *k*-mer (see Supplemental Notes “IgScout pseudocode,” “IgScout parameters,” and “Benchmarking IgScout on simulated immunosequencing datasets”). Figure 2 demonstrates that IgScout reconstructs many known human D genes.

Similarly to the existing tools for reconstructing V and J genes (that typically trim a few nucleotides in the beginning/end of the reconstructed genes), IgScout also trims a few nucleotides in the beginning/end of the reconstructed D genes. Although lowering the *IC* threshold would reduce the number of trimmed nucleotides, we decided not to do it since lowering this parameter may result in erroneous reconstructions and since the trimmed nucleotides hardly affect the downstream applications of IgScout. See Supplemental Note: “How trimmed (rather than complete) D genes affect the downstream analysis of immunosequencing datasets.”

Indeed, the personalized immunogenomics applications [such as the discovery of “deficient” germline variants that lead to poor responses to vaccination (12)] are hardly affected by the fact that all existing tools for inferring the V, D, and J genes trim a few nucleotides from the ends. Reconstruction of monoclonal antibodies from tandem mass spectra and various proteogenomics applications are also hardly affected by this trimming. Moreover, in the case of human germline genes (and other genomes with well-characterized germline genes) the trimmed nucleotides can be tentatively reconstructed based on similarity with known germline genes (as has been done in previous studies of V and J genes). However, in some cases, assigning terminal nucleotides by homology might lead to the inference of erroneous alleles (38–40). Ideally, the gene inference problem should be followed by validation using genomic data that raises need in paired Rep-Seq and WGS datasets from the same individual. The antibody analysis and engineering in model organisms can also be done with partial D genes.

### Limitations and Advantages of IgScout

**Figure 3:**
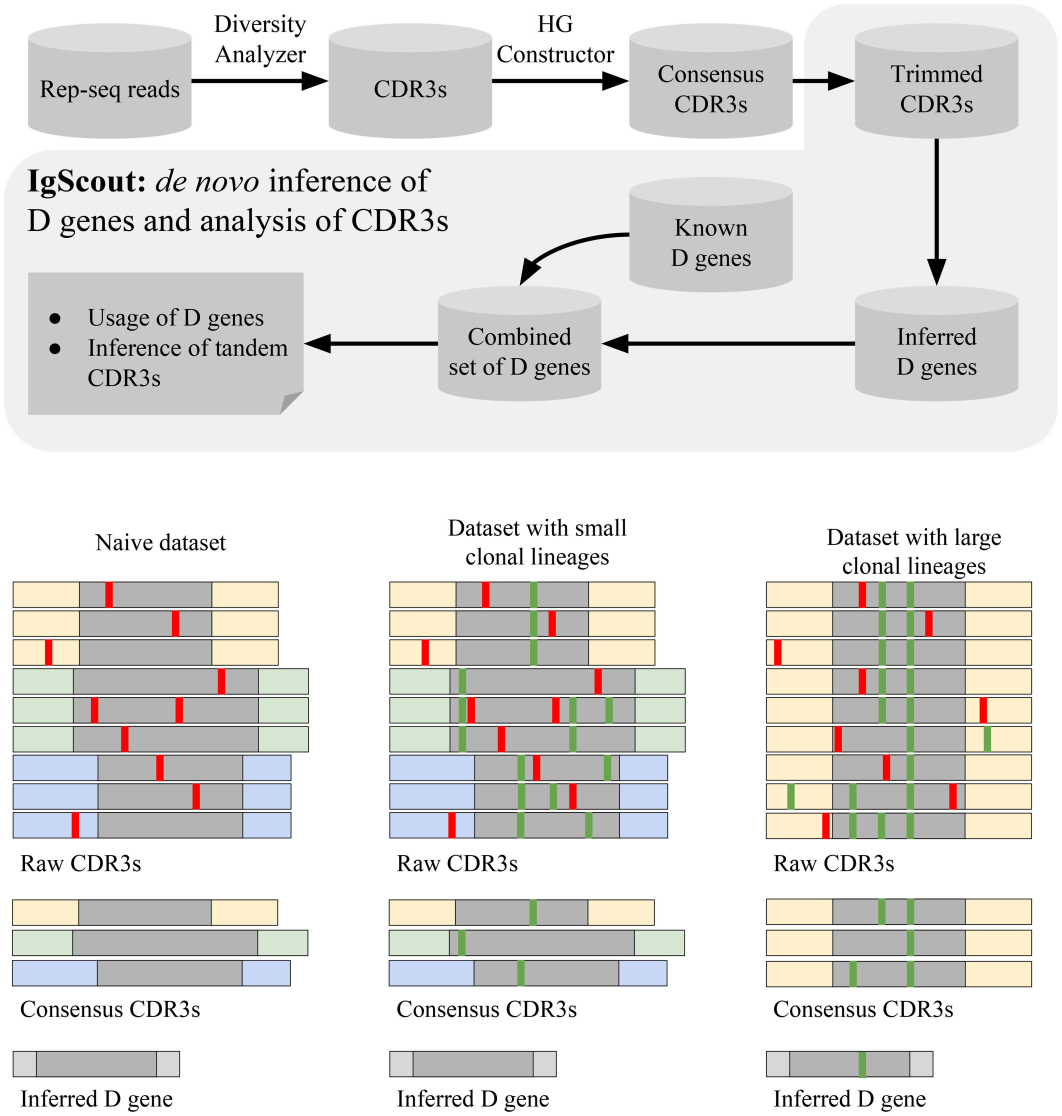
The IgScout pipeline. **(Top)** IgScout steps. **(Bottom)**. IgScout performance on a hypothetical naive dataset (left), a dataset with small clonal lineages **(middle)**, and a dataset with large clonal lineages (right). We assume that all CDR3s are derived from the same D gene (shown in gray). CDR3s corresponding to the same ancestral VDJ recombination are shown by the same color. Sequencing and amplification errors are shown in red; somatic hypermutations are shown in green. The reconstructed (missing) part of the inferred D gene is shown in gray (light gray).

The IgScout pipeline consists of three steps: (i) preprocessing Rep-seq reads; (ii) inferring D genes; (iii) analyzing VDJ recombinations based on the inferred genes (Figure 3). The preprocessing step extracts CDR3s, constructs consensus CDR3s, and trims prefixes and suffixes of CDR3s to exclude suffixes of V genes and prefixes of J genes. The inference step derives D genes from the set of trimmed CDR3s and combines them with the set of known D genes (if available). The final step computes usage of D genes (including analysis of the allele usage of heterozygous D genes) and finds CDR3s with tandem D-D fusions.

Analysis of simulated CDR3s suggests that IgScout correctly reconstructs long D genes (length at least 20 nucleotides) if they give rise to at least 1% of CDR3s but misses short D genes (length < 20 nt) if they give rise to < 2.5% of CDR3s (see Supplemental Note “Benchmarking IgScout on simulated immunosequencing datasets”).

Since it is difficult to distinguish amplification artifacts from SHMs, IgScout takes a conservative approach and partially removes the clonal diversity (step “Hamming Graph (HG) Constructor” in Figure 3) to avoid propagation of amplification errors. Since naïve B cells do not have SHMs, the preprocessing step results in correcting amplification errors and enables reconstruction of long fragments of D genes. As a result, IgScout performs well on datasets with a sufficiently large number of consensus CDR3s (Figure 3). Below we analyze how the number of consensus CDR3s in real datasets affects the IgScout performance.

If a dataset contains hypermutated sequences, then the processing step keep SHMs in the consensus CDR3s. However, if the dataset does not have large clonal lineages (e.g., PBMC from a healthy donor) and the number of consensus CDR3 is large (Figure 3), IgScout treats unremoved SHMs as random errors and still reconstructs mutation-free D genes. However, if a dataset is formed by large clonal lineages, the preprocessing step creates a small number of consensus CDR3s with abundant SHMs. Although IgScout is able to reconstruct some over-represented D genes for such datasets, some of the inferred D genes may still contain SHMs (Figure 3). We thus suggest to use caution while applying IgScout to clonally expanded datasets (see Supplemental Note “How IgScout results are affected by the number of consensus CDR3s and cell types”).

### Reconstruction of Human D Genes

**Figure 4:**
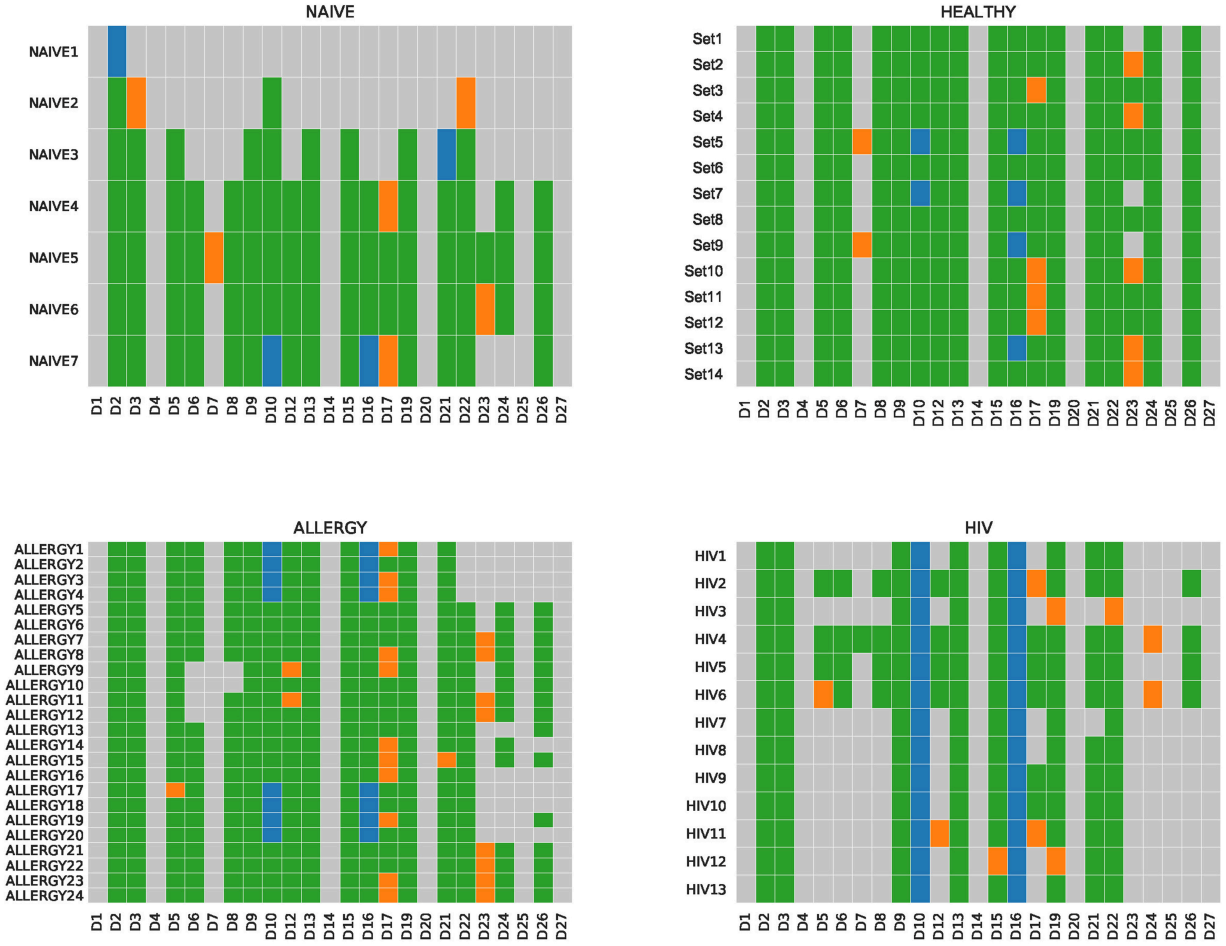
*De novo* reconstructions of D genes across NAÏVE, HEALTHY, ALLERGY, and HIV datasets. Human D genes that were reconstructed (missed) are shown by colored (gray) cells. Green and orange cells correspond to reconstructed D genes listed in the IMGT database. Green cells correspond to substrings of known D genes. Orange cells correspond to substrings that differ from substrings of known D genes by the first or the last nucleotide. Blue cells correspond to novel variants of D10 (IGHD3-10) and D16 (IGHD3-16) genes. For the Set5, Set7, ALLERGY1–ALLERGY4, ALLERGY17–ALLERGY20, HIV1–HIV13, IgScout inferred two variants (novel and known) of D10 (IGHD3-10). The NAIVE datasets are listed in the increasing order of the number of consensus CDR3s in them.

IgScout is best suitable for reconstructing D genes in the case of naive datasets and PBMC datasets with small clonal lineages. To illustrate this point, we applied IgScout to the NAÏVE, HEALTHY, ALLERGY, and HIV datasets. The number of consensus CDR3s in the NAIVE datasets varies from 1,000 to 115,000. Figure 4 shows that IgScout reconstruct the same set of D genes as on the simulated datasets for naïve datasets with at least 20,000 consensus CDR3s. Figure 4 shows that IgScout performs well on the HEALTHY and ALLERGY datasets and reconstructs the same set of D genes as for the simulated and NAÏVE datasets. Since number of consensus CDR3s in some of the HEALTHY and ALLERGY datasets is as low as 40,000, we recommend applying IgScout to dataset with small clonal lineages if the number consensus CDR3s exceeds 40,000. Although the HIV datasets also has many consensus CDR3s (varying from 19,000 to 55,000), the high SHM rate in the HIV datasets makes it difficult to reconstruct some short D genes (Figure 4). We thus suggest to use caution while applying IgScout to highly hypermutated datasets (such as repertoires of HIV and lymphoma patients.

**Figure 5:**
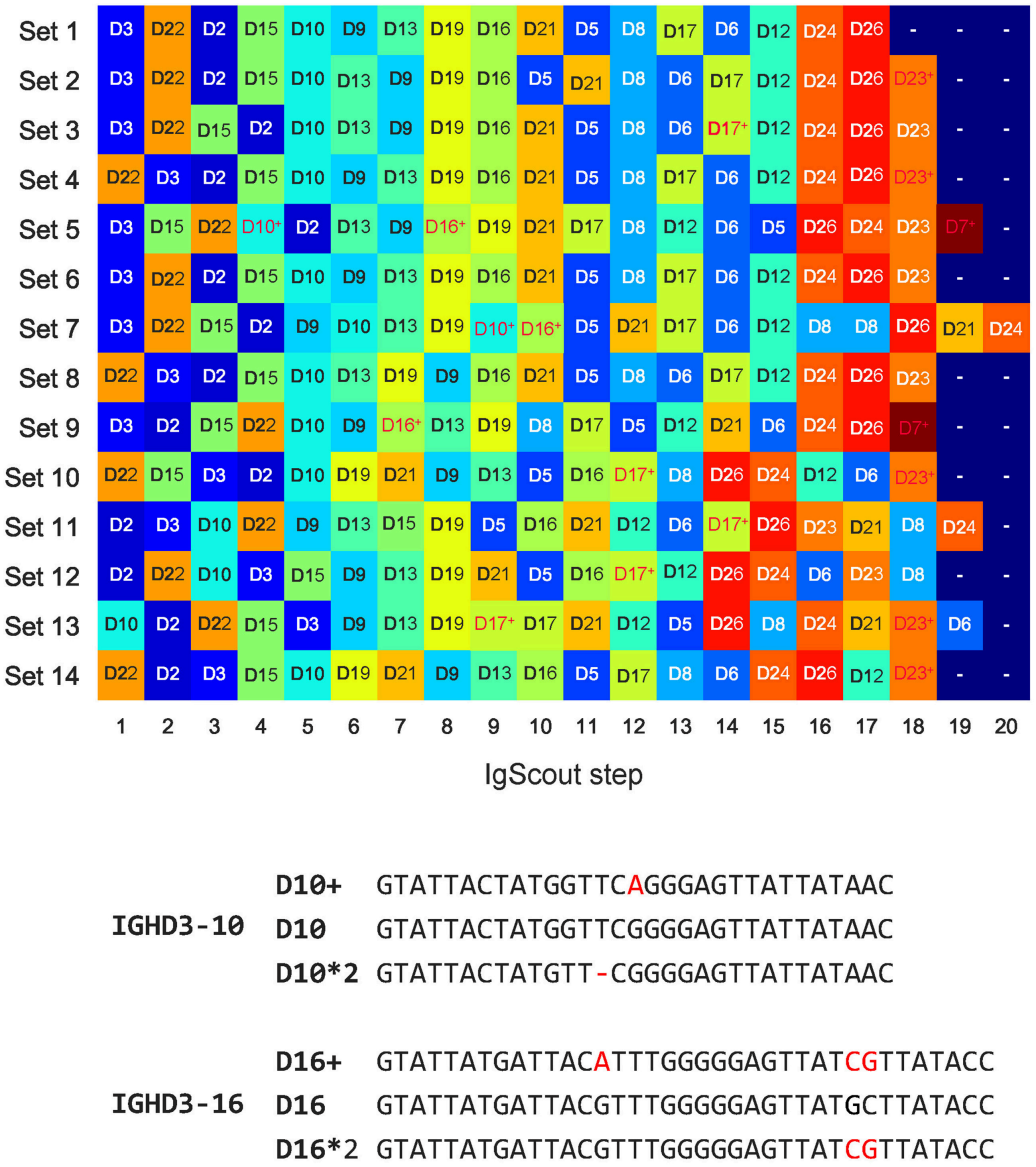
*De novo* reconstructions of D genes across HEALTHY datasets **(top)** and allelic variants D10^+^ and D16^+^ inferred by IgScout **(bottom)**. **(Top)** Genes reconstructed at consecutive steps of IgScout for all HEALTHY datasets. Rows correspond to the datasets and columns correspond to the IgScout steps. Each cell is marked by a reconstructed D gene (each D gene is assigned a unique color). Cells marked with the “+” sign refer to strings that differ from known D genes by at most two nucleotides and correspond to putative novel variants (shown in red). **(Bottom)** Allelic variants D10^+^ and D16^+^ inferred by IgScout. Differences from human D genes and their allelic variants listed in the IMGT database are shown in red.

Figure 5 illustrates that IgScout reconstructed 18 out of 25 human D genes across all HEALTHY datasets, Supplemental Note “Summary of IgScout results across diverse immunosequencing datasets” describes inference of 20 human D genes across multiple immunosequencing datasets. Supplemental Note “Reconstructing variants of human D genes” describes inference of five allelic variants of the D7, D10, D16, D17, and D23 genes, However, since variations in D7, D17, and D23 genes affect the first or last nucleotides of the corresponding D genes, they likely represent computational artifacts caused by abundant nucleotides at the flanking positions of the D genes within CDR3s. In contrast, variations of the D10 and D16 genes (referred to as D10+ and D16+, respectively) have mutations in the middle of D genes (Figure 5). They were inferred from multiple datasets (Set 5 and Set 7 for D10+, and Set 5, Set 7, Set 9, and Set 13 for D16+) and are consistent with alleles identified in previous studies [alleles IGHD3-10^*^p03 and IGV3-16^*^p03 reported in Lee et al. (41) and Boyd et al. (19)], but still missing in IMGT. Supplemental Note “Reconstructing variants of human D genes” illustrates that 50 (42) samples among 600 samples in the PROJECTS10 dataset support D10^+^ (D16^+^) variants and presents two more variants D10^++^ and D16^++^.

To demonstrate that D10+ and D16+ indeed represent new variants of D10 and D16 genes, we analyzed 40 whole genome sequencing datasets from the population-wide study of esophageal cancer (PRJNA427604 project) and searched for exact occurrences of D10+ and D16+ in reads. Both variations were detected in five out of 40 datasets (SRR6435661, SRR6435676, SRR6435686, SRR6435691, and SRR6435692) with the number of reads supporting D10+ (D16+) varying from 8 to 14 (30 to 58) across these five datasets.

In general, IgScout has limitations with respect inferring both variants of a heterozygous D gene. Specifically, if two variants of the same D gene share a *k*-mer and IgScout selects this *k*-mer as a seed, the current version of IgScout may only reconstructs the most abundant variant of this D gene. We plan to enable inference of heterozygous D genes with two novel alleles and thus address this limitation in the next version of IgScout. Currently, to analyze allele usage of heterozygous human D genes, IgScout combines the inferred D genes with known D genes.

### Reconstruction of Camel D Genes

Although camel V genes were inferred in Conrath et al. (43), camel D genes remain unknown. We analyzed six CAMEL datasets from three camels (VH and VHH libraries for each camel) labeled as Camel 1VH, 1VHH, 2VH, 2VHH, 3VH, and 3VHH (33). While the VH libraries contain the heavy chain of the conventional (both heavy and light chain) camel antibodies, the VHH libraries contain the heavy chains of the *single-chain antibodies*.

We extracted camel CDR3s by aligning camel antibody repertoires against the known camel V and J genes using the IgReC tool (34). For the Camel 1VH dataset, IgScout constructed 60,066 consensus CDR3 sequences of average length 48 nucleotides. The CDR3^*^ dataset for Camel 1VH has total length 1,400,360 nucleotides (the average length 23 nt).

IgScout reconstructed four D genes in the case of the Camel 1VH dataset that we refer to as D1, D2, D3, and D4 (see Supplemental Note “Reconstructing camel D genes”). It reconstructed four putative D genes in datasets Camel 1VHH, and Camel 2VH, and three putative D genes in the remaining three camel datasets (17 strings in total) that are largely consistent with genes D1, D2, D3, and D4 derived from the Camel 1VH dataset (previous studies assumed that the camel genome has a single germline D gene (43). Supplemental Note “Reconstructing camel D genes” illustrates that all camel D genes are shared between the VH and VHH datasets. Supplemental Note “Usage of camel D genes” demonstrates that the camel D genes have strikingly different usage in the VH and VHH antibodies.

### D Gene Usage

Twenty-five human D genes form a set of strings that we refer to as *D-Genes*. Given an arbitrary string *Target*, a string *D* from *D-Genes*, and a parameter *k*, we say that a string *Target* is *formed* by *D* if it contains a *k*-mer from *D* but does not contain *k*-mers from other strings in *D-Genes* (the default value *k* = 11). We classify a CDR3 as *traceable* if it is formed by a D gene and *non-traceable*, otherwise. The percentage of traceable CDR3s is rather conservative across all HEALTHY datasets: ≈60% of CDR3s in the HEALTHY datasets are traceable (Supplemental Note “Traceable CDR3s”).

Given a set of strings *Strings* and a string *D* from *D-Genes*, we define *usage*(*Strings, D-Genes, D*) as the fraction of traceable strings in *Strings* formed by the string *D*. We are interested in *usage*(CDR3^*^, *D–Genes, D*) for each human D gene. Supplemental Note “Traceable CDR3s” analyzes the usage of all human D genes across all HEALTHY datasets. Supplemental Note “D gene classification by IgScout and IgBlast” compares IgScout and IgBlast classification of D genes forming CDR3s.

**Figure 6:**
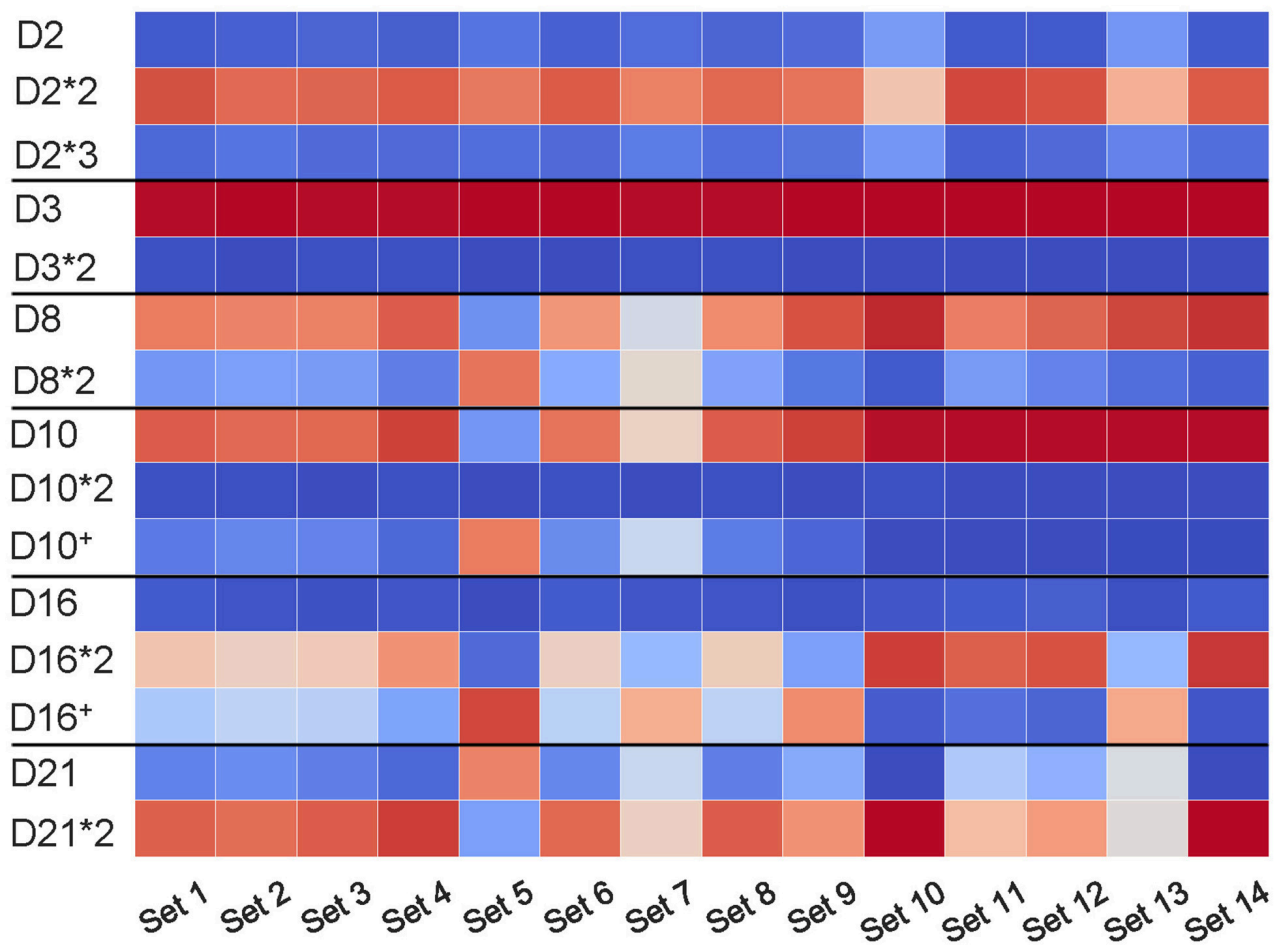
Usage of D genes with known and novel allelic variants across all HEALTHY datasets. Horizontal black lines sub-partition the matrix into six sub-matrices corresponding to allelic variants of D2 (IGHD2-2), D3 (IGHD3-3), D8 (IGHD2-8), D10 (IGHD3-10), D16 (IGHD3-16), and D21 (IGHD2-21). For each D gene and each dataset, we computed the percentage of usage of each variant. Values in cells vary from 0 (blue) to 100 (red). White cells correspond to values ~50% and likely represent cases when a single individual carries different variants of a given D genes on two different chromosomes.

We analyzed the usage of known and novel allelic variants (D10^+^ and D16^+^) across all HEALTHY datasets. Figure 6 reveals that usage of allelic variants of D2 and D3 is consistent across all datasets with D2^*^2 and D3 as dominant variants. However, the Set 5 has different dominant variants as compared to other datasets: D8^*^2 (compared to D8 in all other datasets); D10^+^ (compared to D10 in all other datasets); and D21 (compared to D21^*^2 in all other datasets). The variant D16^+^ is dominant in Sets 5, 7, 9, and 13, while the D16 gene is dominant in the remaining eight datasets.

### Tandem CDR3s

Given strings *D* and *D'*, and a parameter *k*, we say that a string *Target* is *formed* by *D* and *D'* if it contains *k*-mers from both *D* and *D'* and a *k*-mers from *D'* starts after a *k*-mer from *D* ends. Since tandem CDR3s represent a small fraction of all CDR3s, we set the default value *k* = 11 (rather than *k* = 15 for all CDR3s) to increase the number of identified tandem CDR3s. Although a smaller value of *k* may lead to identification of *pseudo-tandem* CDR3s, the Methods section describes how to filter out such pseudo-tandem CDR3s.

**Figure 7:**
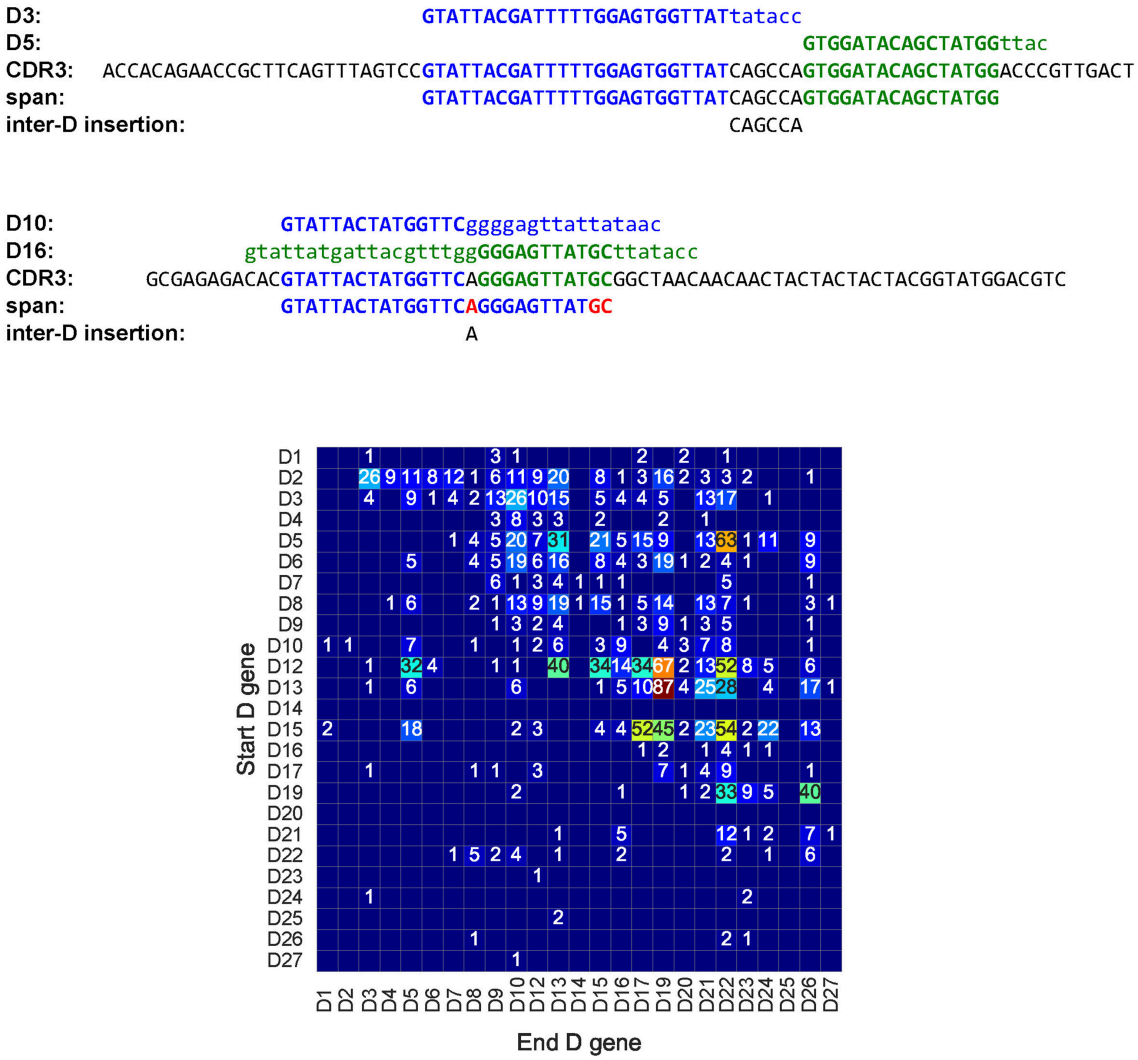
A tandem CDR3 formed by genes D3 (IGHD3-3) and D5 (IGHD5-5) **(top)**, a pseudo-tandem CDR3 formed by genes D10 (IGHD3-10), and D16 (IGHD3-16) **(middle)**, and the tandem matrix for all tandem CDR3s across all HEALTHY datasets **(bottom)**. **(Top)** A tandem CDR3 with *D*_*match*_ = GTATTAGGATTTTTGGAGTGGTTAT, *middle* = CAGCCA, and *D'*_*match*_ = GTGGATACAGCTATGG. **(Middle)** The pseudo-tandem CDR3, formed by genes D10 (IGHD3-10) and D16 (IGHD3-16). This CDR3 was formed by a single gene D10 (IGHD3-10) with three mutations (shown in red). IgScout filters out most pseudo-tandem CDR3s. **(Bottom)** The number in a cell (*i,j*) shows the total number of tandem CDR3s formed by genes *D*_*i*_ and *D*_*j*_ across all HEALTHY datasets. Empty cells correspond to pairs of D genes that do not form tandem CDR3s. Genes D4 and D5 appear in two copies in the IGH loci. The second copy of D4 (IGHD4-11) appears between D10 (IGHD3-10) and D12 (IGHD5-12). The second copy of D5 (IGHD5-18) appears between D17 (IGHD4-17) and D19 (IGHD6-19). The vast majority of tandem CDR3 correspond to cells in the upper half of the matrix. The only populated column in the lower part of the tandem matrix corresponds to the D5 gene and likely results from tandem CDR3s formed by the second copy of D5 in the IGH locus.

There exist 187 *tandem CDR3s* formed by two D genes in the CDR3^*^ dataset (Figure 7). We denote the longest substring between a tandem CDR3 *Target* and *D* (*Target* and *D'*) as *D*_*match*_(*D'*_*match*_) and represent a tandem CDR3 *Target* as a concatenate of five strings *prefix* ${}_{{}^{*}}$ *D*_*match*_ ${}_{{}^{*}}$ *middle* ${}_{{}^{*}}$ *D'*_*match*_ ${}_{{}^{*}}$ *suffix*. We define the *span* of a tandem CDR3 formed by *D* and *D'* as the substring *D*_*match*_ ${}_{{}^{*}}$ *middle* ${}_{{}^{*}}$ *D'*_*match*_ and *inter-D insertion* as the substring *middle* (Figure 7).

Briney et al. (29) emphasized that detecting tandem CDR3s has to be done with caution since they are often confused with *pseudo-tandem* CDR3s formed by the standard V(D)J recombination (Figure 7). The Methods section describes how IgScout detects pseudo-tandem CDR3s. One hundred and fourteen out of 187 tandem CDR3s are not pseudo-tandem in the CDR3^*^ dataset.

### Tandem Bias

There exists 114 tandem CDR3s in the Set 1 dataset and 1900 tandem CDR3s across all HEALTHY datasets. Figure 7 represents all tandem CDR3s as a *tandem matrix* and reveals that the vast majority of them correspond to cells in the upper half of this matrix. If tandem CDR3s were computational artifacts, we would expect similar numbers of CDR3s in the upper and lower parts of the tandem matrix. We define the *tandem bias* as *N*_*lower*_ / (*N*_*upper*_ + *N*_*lower*_), where *N*_*upper*_, and *N*_*lower*_ is the sum of entries in the upper and lower parts of the tandem matrix, respectively (we assume that the main diagonal belongs to the lower part of the matrix). The tandem bias varies from 0.03 to 0.21% across various datasets (see Supplemental Note: “Analysis of tandem CDR3s).

Since most pairs of D genes in tandem CDR3s contribute to the upper part of the tandem matrix (and thus follow the order of D genes in the IGH locus), entries in the lower part of the tandem matrix likely represents false positives. However, some of them may reveal possible duplications of D genes, e.g., the D22 row in the lower part of the tandem matrix in Figure 7 reveals many tandem CDR3s. Analysis of the hepatitis patient 1,776 in the PROJECTS10 dataset (44) revealed particularly many entries in the D22 column in the lower part of the tandem matrix, suggesting a duplication of the D22 gene in this patient (see Supplemental Note “Analysis of tandem CDR3s”). Kidd et al. (23) analyzed biases in the D-J pairing and also suggested that D22 may be duplicated in some individuals.

### Ultra-Long CDR3s Reveal Unusual Recombination Events

One thousand nine hundred tandem CDR3s across all HEALTHY datasets contain 1,081 distinct inter-D insertions, varying in length from 0 to 153 nucleotides. The two longest inter-D insertions (denoted *I*_1_ and *I*_2_) appear in the Set 1 and have length 153 nucleotides. They are formed by genes D9 and D10, differ by a single nucleotide, and appear in CDR3s differing by six nucleotides. Surprisingly, the inter-D insertion *I*_2_ coincides with the sequence of the IGH locus between the D9 and D10 genes. Germline D genes are flanked by *recombination signal sequences* (*RSSs*) with 12-nucleotide long spacer and the inter-D insertion *I*_2_ starts with the right RSS of D9 and ends with the left RSS of D10 (Supplemental Note “Ultra-long tandem CDR3s”).

Thus, ultra-long tandem CDR3s reveal unusual *RSS skipping* events during somatic recombination: skipping the right RSS of D9 and left RSS of D10 led to a tandem CDR3 representing a concatenate D9 + *I*_2_ + D10. Although the found example is not productive, we also detected RSS skipping in nine productive ultra-long CDR3s across all HEALTHY and ALLERGY datasets. All productive CDR3s are formed by skipping of the right RSS of D22. Instead of it, somatic recombination uses a cryptic RSS (CACAGCA + ACCCAAACA) located at the distance 129 nt from the end of D22 and forms ultra-long CDR3s containing a genomic fragment of the IGH locus that starts with the right RSS of D22 (Supplemental Note “Ultra-long CDR3s”). The discovery of productive ultralong CDR3s challenges the conventional view of germline genes as non-overlapping substrings of DNA and reveals the first example of *nested* D genes, when one D gene is contained within another D gene.

The existing immunosequencing protocols are likely to miss ultra-long immunoglobulins since they are not designed to capture the abnormally long variable regions (exceeding ~400 nt). We captured reads containing ultra-long tandem CDR3s because the 300-nucleotide long paired reads (overlapping by only 50 nucleotides) in the Set 1 and ALLERGY datasets are longer than reads used in most other immunosequencing datasets. Thus, even if ultra-long tandem CDR3s were common, they would likely remain below the radar of most immunosequencing studies.

### Tandem CDR3s Contribute to Adaptive Immune Response

We investigated whether tandem CDR3s contribute to the adaptive immune response by analyzing their *isotypes*. Since IgG, IgA, and IgE isotypes occur in plasma and memory B cells subjected to the antibody-antigen interactions, these isotypes they indicate (in difference from IgM isotypes common in memory and naïve B cells) that the corresponding antibodies participate in the adaptive immune response.

**Figure 8:**
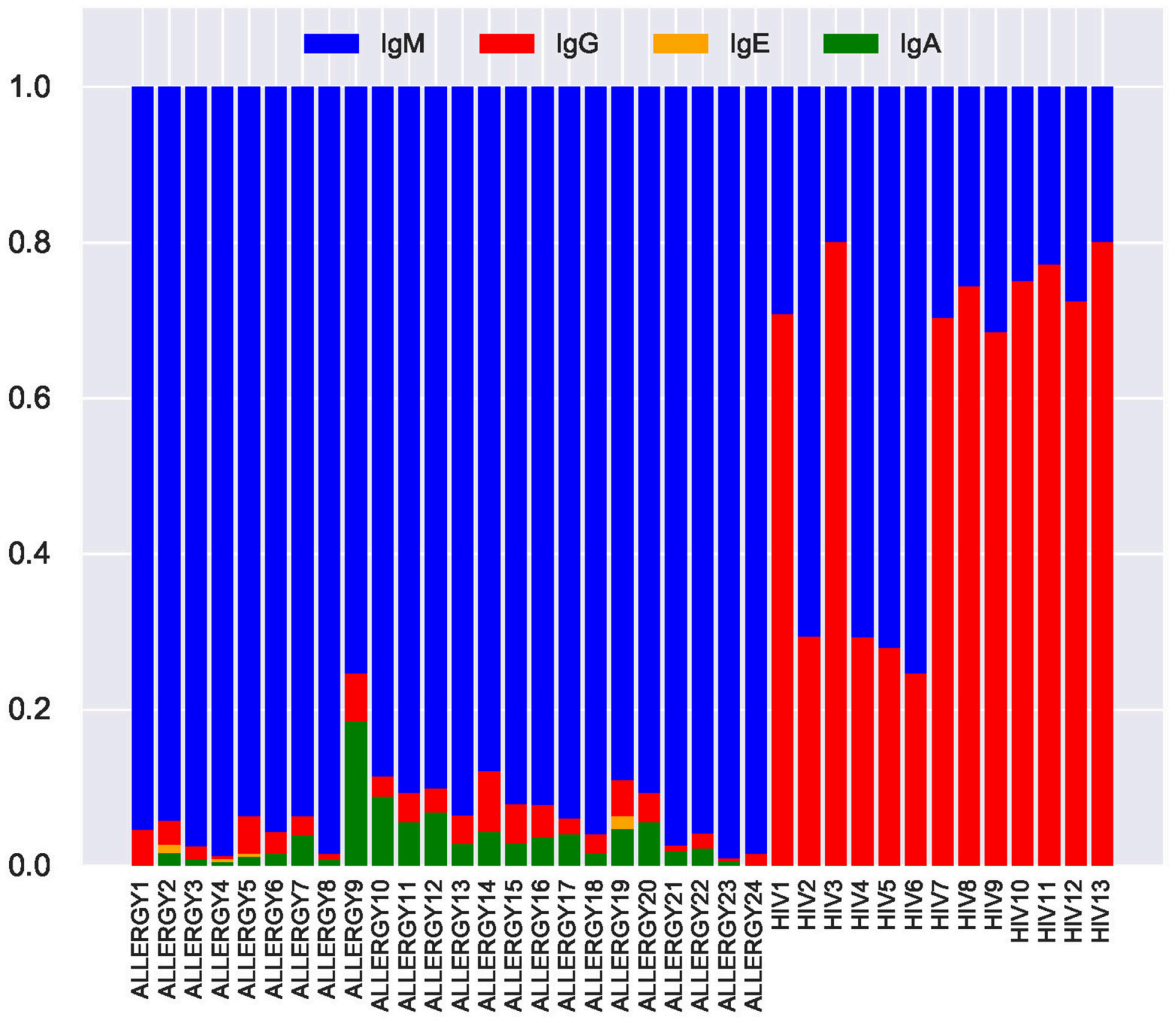
Fractions of IgM, IgG, IgE, and IgA isotypes representing tandem CDR3s in the repertoires from the ALLERGY and HIV datasets.

We inferred isotypes in the ALLERGY and HIV datasets using markers described in Levin et al. (31) (Figure 8). The vast majority of tandem CDR3s from the ALLERGY dataset correspond to the IgM isotype and thus are produced by memory and naïve B cells. In contrast, ~60% of tandem CDR3s in the HIV dataset correspond to the IgG type. This observation suggests that tandem CDR3s in the HIV-infected patients arise from immunoglobulins that are produced by plasma cells and thus might contribute to the immune response against HIV antigens.

## Discussion

Since many human germline alleles remain unknown (particularly for non-European subjects), missing alleles may mislead clinical decisions (45) and lead to erroneous derivation of clonal lineages due to misinterpretations of SHMs. Thus, finding new germline alleles and building personalized sets of germline genes for each individual is important for downstream analysis of immunosequencing datasets.

Although there exists a number of tools for inferring V and J genes (3, 21, 22), a more difficult problem of reconstructing D genes remains open. IgScout aims to reconstruct all D genes explaining a large percentage of the VDJ recombination in an antibody repertoire rather than to reconstruct all D genes. The IMGT database reflects the *genomic diversity* of D genes but not their *recombinant diversity* (information about rearrangements, transcription, and translation of D genes). Since assemblies of the highly repetitive IGH loci are fragmented and error-prone (7, 14, 42, 46) reconstruction of all germline genes from the whole-genome sequencing data is a difficult problem. Although the IGH locus is extremely diverse (16), it remains largely unknown how it varies across the human population. Moreover, even in the case when the IGH locus is correctly assembled, prediction of the functional germline genes is a non-trivial problem (2, 13).

Immunosequencing datasets reflect the recombinant diversity of antibody repertoires and thus complement the genomic datasets. If some D genes do not contribute to the VDJ recombination (e.g., our analysis suggests that genes D1, D14, D20, D25, and D27 do not significantly contribute to VDJ recombination in any of the analyzed datasets), they have limited contribution to immune response. In this paper, we focused on reconstructing D genes shaping the recombinant diversity rather than all D genes.

IgScout reconstructed 20 out of 25 human D genes across multiple datasets and missed genes D1, D14, D20, D25, D27 that form a small number of CDR3s (< 0.1% each) across all analyzed datasets. It remains unclear whether some of these genes ever contribute to any CDR3s, for example genes D14 and D25 do not form any CDR3s in most datasets (few CDR3s formed by these D genes in some datasets may represent computational artifacts).

IgScout revealed four new allelic variants (D10^+^, D10^++^, D16^+^, and D16^++^), thus increasing the number of known variants of human D genes from 7 to 11. These new variants are unlikely to be computational artifacts since they were found in dozens immunosequencing datasets from distinct individuals and many whole genome sequencing datasets. The frequency of the already known Single Nucleotide Polymorphisms (SNPs) in D genes exceeds the frequency of SNPs in the entire human genome by two orders of magnitude (12 SNPs for all D genes of total length only 288 nucleotides).

Although IgScout revealed four novel variants of human D genes and inferred camel D genes, these genes will not be included in the IMGT database since they haven't been experimentally confirmed yet. Similarly to Gadala-Maria et al. (20), we argue that, like in other areas of genomics, the time has come to add such prediction to the IMGT database. For example, the lion's share of genes in genomic databases represent computational predictions that haven never been experimentally confirmed. We argue that IMGT should classify alleles with varying levels of supporting evidence, not unlike classification systems used in other biological databases and in the recently established Open Germline Receptor Database (OGRDB), a new repository of germline genes maintained by The Adaptive Immune Receptor Repertoire (AIRR) Community (47).

Although IgScout is not specifically designed for reconstructing V and J genes, it turned out that its applications are not limited to reconstructing D genes (see Supplemental Note “*De novo* reconstruction of human J genes”). In addition to *de novo* reconstruction of D genes, it also detects tandem CDR3s. Briney et al. (29) postulated that tandem CDR3s mostly appear in naïve B cells and thus do not contribute to adaptive immune response. In contrast, our analysis revealed that ~60% of tandem CDR3s in the HIV dataset correspond to plasma and memory B cells.

## Methods

### Inferring Germline Genes as the Trace Reconstruction Problem

In information theory, a string *S* yields a collection of *traces*, where each trace is independently obtained from *S* by substituting each symbol in *S* by another symbol from a fixed alphabet with a given probability δ. Given the traces and the value δ, the *Trace Reconstruction Problem* (35) is to reconstruct the original string *S*. *De novo* reconstruction of D genes results in a more complex version of the Trace Reconstruction Problem where traces are generated by multiple strings and each trace is obtained from one of these strings by (i) randomly trimming it from both sides, (ii) adding a randomly generated prefix in the front of the string, and (iii) adding a randomly generated suffix in the end of the string. Given a set of such traces (modeled by a set of trimmed CDR3s extracted from an immunosequencing dataset), the goal is to reconstruct the original set of strings.

### Extending the Consensus String

IgScout trims all positions of the motif logo with the information content below *IC* and computes the consensus string. Afterwards, it extracts the first *k*-mer of the consensus string and finds all CDR3s that contain this *k*-mer. If the position preceding the first *k*-mer in these reads has information content exceeding a threshold, IgScout adds the most frequent nucleotide at this position to the consensus and iterates. Afterwards, it applies a similar procedure to the last *k*-mer of the consensus string. The resulting extended consensus is reported as a putative D gene (Figure 1).

### Detecting Pseudo-Tandem CDR3s

Given strings *Span* and *S*, we define *distance*_*t*_(*Span,Target*) as the minimum Hamming distance between *t*-mers in *Span* and *S*. Given a parameter Δ (the default value Δ = 5) we define the Δ*-distance* between strings *Span* and *Target* as *distance*_*t*_*(S,Target*) for *t* = *|Span|*-Δ, where |*Span*| stands for the length of the string *Span*. Finally, we define the Δ*-distance* between a string *Span* and a set of strings *Strings* as the minimum Δ-distance between *Span* and all strings in *Strings*.

We computed the Δ-distance between the spans of all 187 identified tandem CDR3s in CDR3^*^ and all string in *D-Genes*. Seventy-three out of these 187 CDR3s can be explained as CDR3s originating from a single D gene (for the Δ-distance threshold three). However, the remaining 114 CDR3s have Δ-distance at least nine. We thus classify a CDR3 sequence *Target* formed by genes *D* and *D'* as pseudo-tandem if the Δ-distance between the span of this pseudo-tandem CDR3 and *D-Genes* does not exceed a predefined threshold (the default value is three), and (truly) tandem, otherwise. See Supplementary Note “List of tandem CDR3s.”

## Author Contributions

YS implemented the IgScout algorithm and performed benchmarking. YS and PP conceived the study, developed the IgScout algorithm, designed the computational experiments, and wrote the manuscript.

### Conflict of Interest Statement

The authors declare that the research was conducted in the absence of any commercial or financial relationships that could be construed as a potential conflict of interest.
