# Supplementary material for: De novo Inference of Diversity Genes and Analysis of Non-canonical V(DD)J Recombination in Immunoglobulins
Source: Front Immunol. 2019 May 3;10:987. doi: 10.3389/fimmu.2019.00987 (PMC6516046; doi:10.3389/fimmu.2019.00987)
Supplement: Supplementary file 1 [file Data_Sheet_1.pdf]

Supplemental Notes

Immunosequencing datasets

Preprocessing CDR3 datasets

Information about human D genes

Common k-mers

Analysis of inverted D genes

IgScout pseudocode

IgScout parameters

Simulating CDR3 datasets

Benchmarking IgScout on simulated immunosequencing datasets

How trimmed (rather than complete) D genes affect the downstream analysis of immunosequencing datasets

Reconstructing variants of human D genes

Summary of IgScout results across diverse immunosequencing datasets

How IgScout results are affected by the number of consensus CDR3s and cell types

Reconstructing camel D genes

Usage of camel D genes

Traceable CDR3s

D gene classification by IgScout and IgBlast

Analysis of tandem CDR3s

Ultra-long CDR3s

De novo reconstruction of human J genes

List of tandem CDR3s

Supplemental Note: Immunosequencing datasets

We analyzed the following immunosequencing datasets:

- HEALTHY: 14 datasets from PBMC of healthy individuals labeled as Set 1 – Set 14 available from the NCBI projects PRJNA395083 and PRJNA324093 (Table A1).
- ALLERGY: 24 datasets from PBMC and bone marrow of allergy patients available from the NCBI project PRJEB18926 and labeled as ALLERGY 1 – ALLERGY 24 (Table A2).
- HIV: 13 datasets from PBMC of HIV-infected patients available from the NCBI project PRJNA396773 and labeled as HIV 1 – HIV 13 (Table A3).
- NAÏVE: 7 datasets from naïve B cells available from the NCBI projects PRJNA324093 and PRJNA355402 (Table A4).
- PROJECTS10: 600 datasets from various human subjects and various tissues corresponding to ten NCBI projects (Table A5).
- CAMEL: 6 datasets from PBMC of healthy camels labeled as Camel 1VH, Camel 1VHH, Camel 2VH, Camel 2VHH, Camel 3VH, and Camel 3VHH (Table A6).

All analyzed datasets are sequenced from RNA sources. Datasets Set 1 – Set 9 were generated in Dr. Chudakov's lab at Moscow Institute for Bioorganic Chemistry to study aging of the adaptive immune system. Datasets Set 10 – Set 14 were generated to study immunological response to vaccines (Ellebedy et al., 2016). These datasets contain heavy chain repertoires extracted from peripheral blood mononuclear cells (PBMC) of fourteen healthy individuals. Although B cells from peripheral blood contain SHMs, we do not expect to see large clonal lineages in healthy donors.

Each analyzed dataset contains at least 40,000 distinct VDJ recombination events (corresponding to approximately million paired-end reads). Reduction in the dataset size (e.g., from a million to 100,000 reads) negatively affects the performance of IgScout and makes it difficult to capture tandem CDR3s.

In addition to the example of ultra-long CDR3 provided in the paper, we also detected RSS skipping between D9 and D10 in the ALLERGY datasets and added information about them to Supplemental Note "Ultra-long tandem CDR3s". In the future, we plan to investigate the genomic insertions in ultra-long CDR3s using high-throughput Rep-seq data from the latest studies (Soto et al., 2019; Briney et al., 2019) in a separate paper.

dataset	accession number	# reads	# distinct CDR3s	# consensus CDR3s	# trimmed CDR3s
Set 1	SRR8892051	1,611,497	228,619	98,576	82,653
Set 2	SRR8892052	1,497,830	226,206	93,561	75,472
Set 3	SRR8892059	783,971	80,741	39,930	33,123
Set 4	SRR8892053	1,231,238	176,250	111,752	95,278
Set 5	SRR8892054	1,213,516	218,157	141,518	118,862
Set 6	SRR8892055	2,062,940	209,257	90,465	75,978
Set 7	SRR8892056	2,263,605	277,715	152,999	124,837
Set 8	SRR8892057	1,748,496	163,215	80,212	67,382
Set 9	SRR8892058	1,392,370	256,232	153,251	132,595
Set 10	SRR3620050	1,309,906	379,695	129,162	102,768
Set 11	SRR3620092	613,907	181,511	102,186	84,430
Set 12	SRR3620100	599,674	184,143	112,820	115,005
Set 13	SRR3620109	602,833	213,507	158,332	130,560
Set 14	SRR3620118	497,441	212,070	144,299	119,731

Table A1. Information about the HEALTHY immunosequencing datasets. The "# distinct CDR3s" column refers to the number of distinct CDR3s extracted from reads. The "# consensus CDR3s" column refers to the number of distinct consensus CDR3s. The "# trimmed CDR3s" column shows the number of trimmed CDR3s that are longer than k (the default value k = 15). Some of the listed datasets are in the process of uploading to SRA.

dataset	accession number	# reads	# distinct CDR3s	# consensus CDR3s	# trimmed CDR3s
**Donor 1**					
ALLERGY1	ERR1812282	1,249,203	213,573	104,981	89,215
ALLERGY2	ERR1812283	1,566,025	292,102	160,637	137,836
ALLERGY3	ERR1812288	1,782,715	291,796	173,419	150,577
ALLERGY4	ERR1812289	1,372,999	263145	172,202	149,626
**Donor 2**					
ALLERGY5	ERR1812284	1,313,874	353,957	189,172	163,373
ALLERGY6	ERR1812285	1,578,854	411,139	227,437	196,932
ALLERGY7	ERR1812290	644,711	185,269	133,524	113,985
ALLERGY8	ERR1812291	1,208,581	259,113	173,590	148,412
**Donor 3**					
ALLERGY9	ERR1812286	1,260,585	174,620	72,466	62,208
ALLERGY10	ERR1812287	2,366,528	270,805	95,473	81,552
ALLERGY11	ERR1812292	2,116,149	350,726	184,033	157,660
ALLERGY12	ERR1812293	1,842,407	308,770	167,897	143,617

**Donor 4**					
ALLERGY13	ERR1812294	1,935,709	225,119	98,917	83,730
ALLERGY14	ERR1812295	1,526,356	207,544	101,642	85,928
ALLERGY15	ERR1812300	783,249	174,985	129,828	108,518
ALLERGY16	ERR1812301	1,107,910	228,960	169,861	142,102
**Donor 5**					
ALLERGY17	ERR1812296	1,426,885	269,077	125,283	108,452
ALLERGY18	ERR1812297	2,140,711	390,376	190,216	166,223
ALLERGY19	ERR1812302	942,524	110,675	57,414	47,864
ALLERGY20	ERR1812303	1,383,359	118,322	52,666	42,870
**Donor 6**					
ALLERGY21	ERR1812298	2,349,277	391,293	203,739	175,067
ALLERGY22	ERR1812299	2,137,156	357,480	187,634	160,635
ALLERGY23	ERR1812304	1,018,489	183,855	114,532	97,957
ALLERGY24	ERR1812305	818,062	136,659	81,853	69,686

Table A2. Information about the ALLERGY immunosequencing datasets. The first two datasets within each group represent the bone marrow samples (BM) and the second two datasets represent the peripheral blood samples (PBMC). For example, for the datasets ALLERGY 1 and ALLERGY 2 represent BM samples and the datasets ALLERGY 3 and ALLERGY 4 represent PBMC samples.

dataset	accession number	# reads	# distinct CDR3s	# consensus CDR3s	# trimmed CDR3s
HIV1	SRR5888724	775,005	128,433	26,887	21,696
HIV2	SRR5888725	1,961,141	246,330	55,271	42,302
HIV3	SRR5888726	893,865	115,323	25,235	19,981
HIV4	SRR5888727	1,914,113	241,801	54,492	41,689
HIV5	SRR5888728	1,666,263	200,016	45,763	35,284
HIV6	SRR5888729	1,896,887	215,481	47,593	36,450
HIV7	SRR5888730	812,033	124,228	24,200	18,677
HIV8	SRR5888731	1,446,869	155,623	30,938	25,815
HIV9	SRR5888732	1,856,458	198,851	39,164	32,349
HIV10	SRR5888733	1,138,382	115,075	28,911	24,168
HIV11	SRR5888734	1,371,172	145,683	32,407	26,809
HIV12	SRR5888735	1,460,715	128,252	19,334	16,503
HIV13	SRR5888736	1,485,469	108,508	25,021	20,506

Table A3. Information about the HIV immunosequencing datasets.

dataset	accession number	# reads	# distinct CDR3s	# consensus CDR3s	# trimmed CDR3s
NAIVE1	SRR3620104	51,895	4236	1023	1023
NAIVE2	SRR3620095	68,618	7322	1879	1879
NAIVE3	SRR3620054	40,722	9693	7693	7693
NAIVE4	SRR3620035	90,001	31,180	20,147	20,147
NAIVE5	SRR5063092	541,016	119,070	50,991	50,991
NAIVE6	SRR5063097	391,924	164,061	98,887	98,887
NAIVE7	SRR5063084	437,530	175,230	115,140	115,140

Table A4. Information about the NAÏVE immunosequencing datasets.

NCBI project	Reference	# datasets
PRJEB18926	Levin et al., 2017	24
PRJNA396773	Landais et al., 2017	13
PRJNA308641	Galson et al., 2015	107
PRJNA324093	Ellebedy et al., 2016	95
PRJNA248475	Stern et al., 2014	32
PRJNA308566	Galson et al., 2016	142
PRJNA355402	Magri et al., 2017	93
PRJNA393446	Waltari et al., 2018	42
PRJNA349143	Gupta et al., 2017	24

PRJNA430091	Friedensohn et al., 2018	28

Table A5. Information about the PROJECTS10 immunosequencing datasets. The "# datasets" column shows the number of datasets in each project.

dataset	accession number	# reads	# distinct CDR3s	# consensus CDR3s	# trimmed CDR3s
Camel 1VH	SRR3544217	369,502	183,973	60,006	46,326
Camel 1VHH	SRR3544218	339,758	157,938	43,880	39,701
Camel 2VH	SRR3544219	288,099	170,899	74,368	58,118
Camel 2VHH	SRR3544220	281,403	164,087	74,846	68,808
Camel 3VH	SRR3544221	347,291	176,854	79,382	61,918
Camel 4VHH	SRR3544222	343,485	150,724	59,322	53,799

Table A6. Information about the CAMEL immunosequencing datasets.

Supplemental Note: Preprocessing CDR3 datasets

Although IgScout performs a more aggressive error correction (clustering CDR3s that differ by at most 3 mismatches) than the error correction implemented in IgReC (Shlemov et al., 2017) repertoire construction tool, the resulting consensus CDR3s may still contain amplification errors. However, these errors do not corrupt our analysis since they typically result in the low abundance *k*-mers that are not considered by IgScout.

To exclude suffixes (prefixes) of V (J) genes from the constructed set of CDR3s, we trimmed prefixes (suffixes) of CDR3s if they represent suffixes of V genes (prefixes of J genes). If fragments of known V and J genes were not found in a CDR3, we nevertheless cropped it by 10 nucleotides from the start (the end) to remove suffixes (prefixes) of mutated or still unknown V and J genes.

Supplemental Note: Information about human D genes

All human D genes are located in a 30 kb long region in the human IGH locus. Figure A1 shows allelic variants of human D genes listed in the IMGT database.

```
D2     AGGATATTGTAGTAGTACCAGCTGCTATGCC      D10    GTATTACTATGGTTCGGGGAGTTATTATAAC
D2*2   AGGATATTGTAGTAGTACCAGCTGCTATACC      D10*2  GTATTACTATG-TTCGGGGAGTTATTATAAC
D2*3   TGGATATTGTAGTAGTACCAGCTGCTATGCC

D3     GTATTACGATTTTTGGAGTGGTTATTATACC      D16    GTATTATGATTACGTTTGGGGGAGTTATGCTTATACC
D3*2   GTATTAGCATTTTTGGAGTGGTTATTATACC      D16*2  GTATTATGATTACGTTTGGGGGAGTTATCGTTATACC

D8     AGGATATTGTACTAATGGTGTATGCTATACC      D21    AGCATATTGTGGTGGTGATTGCTATTCC
D8*2   AGGATATTGTACTGGTGGTGTATGCTATACC      D21*2  AGCATATTGTGGTGGTGACTGCTATTCC
```

Figure A1. Allelic variants of human D genes listed in the IMGT database. Differences between various variants are highlighted in red. Alleles of human D genes differ from the main variants in a single mutation (D2*2, D2*3, D10*2, D16*2, and D21*2) or two mutations at adjacent positions (D3*2 and D8*2).

We say that two *k*-mers are *δ-similar* if the Hamming distance between them does not exceed the parameter δ. To evaluate similarities between D genes, we constructed their *similarity graph* $SimGraph_{k,\delta}$ in which each D gene corresponds to a vertex and two vertices are connected by an edge if the corresponding D genes contains δ-similar *k*-mers. The weight of the edge connecting two D genes in the similarity graph is defined as the number of δ-similar *k*-mers between these genes.

Figure A2 shows non-trivial connected components of the similarity graphs $SimGraph_{15,1}$ and $SimGraph_{15,2}$ for human D genes and illustrates that connected components contain D genes from the same family of D genes.

Since the similarity graphs $SimGraph_{15,1}$ and $SimGraph_{15,2}$ for camel D genes are empty, we reduced the parameter k from 15 to 8 and constructed the graph $SimGraph_{8,2}$ for camel D genes. As Figure A2 (left) illustrates, all four camel D genes are similar to each other with respect to shared 8-mers.

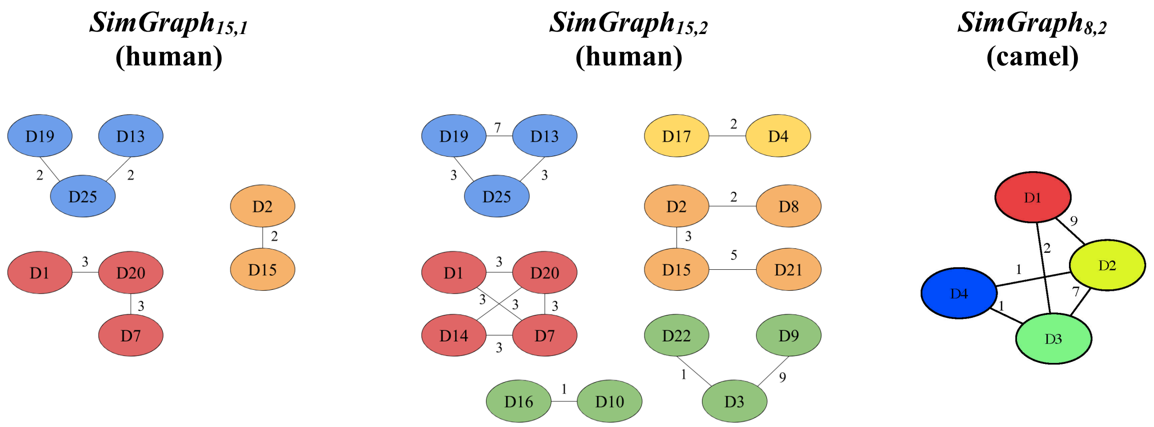

Figure A2. Non-trivial connected components in the similarity graphs $SimGraph_{15,1}$ for human (left), $SimGraph_{15,2}$ for human (middle), and $SimGraph_{8,2}$ for camel (right) D genes. We assigned an individual color to each family of human D genes and to each camel D gene. The graph $SimGraph_{15,4}$ consists of a single connected component containing all human D genes. Human D genes form seven gene families: F1 shown in red: D1 (D1-1), D7 (D1-7), D17 (D1-14), D20 (D1-20), D26 (D1-26); F2 shown in orange: D2 (D2-2), D8 (D2-8), D15 (D2-15), D21 (D2-21); F3 shown in green: D3 (D3-3), D9 (D3-9), D10 (D3-10), D16 (D3-16), D22 (D3-22); F4 shown in yellow: D4 (D4-4), D17 (D4-17), D23 (D4-23); F5: D5 (D5-5), D12 (D5-12), D24 (D5-24); F6 shown in blue: D6 (D6-6), D13 (D6-13), D19 (D6-19), D25 (D6-25); F7: D27 (D7-27).

Supplementary Note: Common *k*-mers

Table A7 provides information about common 15-mers in all HEALTHY datasets. Figure A3 illustrates that most 15-mers from human D genes have high abundances and low ranks.

The proposed classification of *k*-mers reports *k*-mers originated from reverse complemented D genes (referred to as *inverted* *k*-mers) as foreign *k*-mers (unless they are classified as known, mutated, or trimmed). Analysis of inverted CDR3s is described in Supplemental Note “Analysis of inverted D genes”.

dataset	known 15-mers		mutated 15-mers		trimmed 15-mers		foreign 15-mers	
	# (%)	min / max	# (%)	min / max	# (%)	min / max	# (%)	min / max
Set 1	175 (40)	83 / 3141	195 (44)	83 / 645	70 (15)	83 / 604	3 (1)	83 / 134
Set 2	174 (40)	77 / 2850	185 (43)	76 / 587	68 (16)	76 / 556	3 (1)	94 / 104
Set 3	174 (43)	34 / 1070	165 (41)	34 / 222	63 (15)	34 / 199	2 (1)	35 / 41
Set 4	177 (38)	99 / 3921	193 (42)	96 / 739	83 (18)	96 / 728	7 (2)	96 / 131
Set 5	169 (39)	120 / 4699	159 (37)	119 / 2252	82 (19)	119 / 1204	22 (5)	121 / 252
Set 6	176 (43)	76 / 2483	173 (42)	76 / 547	59 (14)	77 / 520	3 (1)	91 / 114
Set 7	174 (45)	128 / 4313	143 (37)	126 / 1001	64 (17)	126 / 877	2 (1)	129 / 130
Set 8	168 (42)	72 / 2371	168 (41)	68 / 523	65 (16)	68 / 491	3 (1)	70 / 98
Set 9	180 (34)	134 / 6505	234 (44)	133 / 1877	106 (20)	136 / 1728	8 (2)	135 / 278
Set 10	180 (38)	104 / 4627	193 (41)	103 / 1319	89 (19)	103 / 763	6 (2)	112 / 185
Set 11	176 (42)	86 / 4007	163 (38)	85 / 890	80 (19)	86 / 513	4 (1)	85 / 131
Set 12	176 (42)	121 / 5241	162 (39)	116 / 1094	75 (18)	116 / 675	3 (1)	122 / 217
Set 13	177 (40)	134 / 4663	176 (40)	131 / 1143	80 (18)	131 / 1066	5 (2)	135 / 175
Set 14	175 (38)	123 / 5650	182 (40)	120 / 1309	93 (20)	120 / 1084	8 (2)	122 / 183

Table A7. Information about known, mutated, trimmed, and foreign *k*-mers among common 15-mers across all HEALTHY datasets. The “# (%)” columns show the number (percentage) of 15-mers of each type. The “min / max” columns refer to the minimal / maximal abundance of 15-mers of each type.

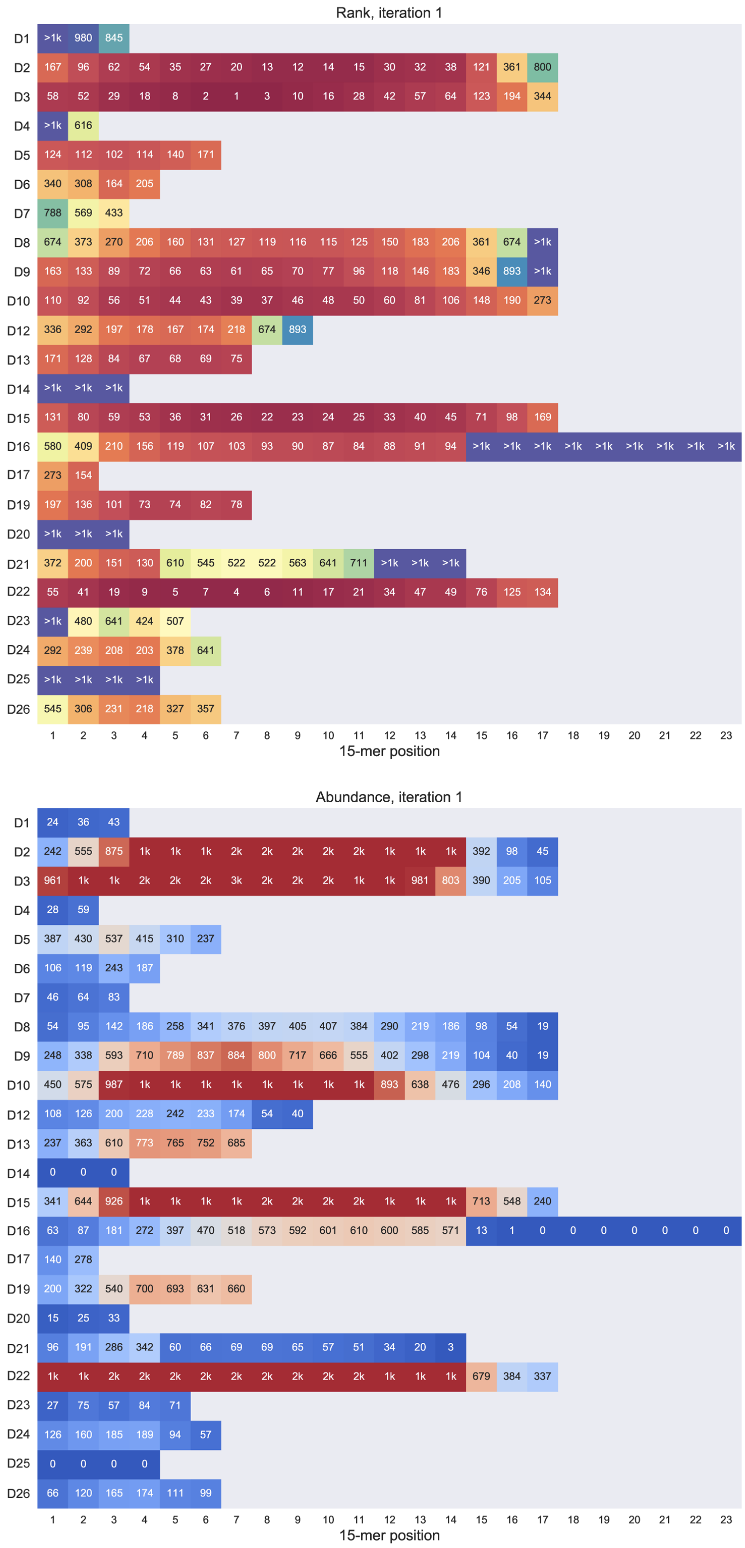

Figure A3. Ranks (top) and abundances (bottom) of 15-mers from human D genes computed for the *CDR3 dataset.** Each D gene of length t is shown as a sequence of t-14 cells representing its 15-mers. For example, the gene D1 of length 17 is shown as a sequence of 3 cells. A number within a cell represents the rank (top) or abundance (bottom) of the corresponding 15-mer. For example, D3 contains the most abundant 15-mer with rank 1 and abundance 3141. Red (blue) cells correspond to low (high) values of ranks and high (low) values of abundances. 11-nucleotide long gene D27 is not shown. Genes D14, D25 and D27 do not contribute 15-mers to the *CDR3** dataset.

Supplemental Note: Analysis of inverted D genes

Since IGHD genes are flanked by RSSs from both sides, Meek et al., 1989 hypothesized that some CDR3s are formed by inverted D genes and identified few inversions in mouse immunoglobulins using D gene primers. Since the classification of *k*-mers proposed in the main text reports *k*-mers originated from reverse complemented D genes (referred to as *inverted k-mers*) as foreign *k*-mers (unless they are classified as known, mutated, or trimmed), we computed a fraction of inverted *k*-mers in foreign *k*-mers.

For each D gene and each dataset, we computed the *inversion coefficient* as the ratio of the average multiplicity of all its inverted 15-mers to the average multiplicity of all its direct 15-mers. Only five D genes (D2, D9, D13, D19, and D22) have non-zero inversion coefficients in at least 10 out of 14 HEALTHY datasets (Figure A4). The average values of the inversion coefficients for these genes vary from 0.001 to 0.007, suggesting that the frequency of D gene inversions may vary between 1 per 200 to 1 per 1000 VDJ recombinations. However, further analysis of statistical significance of this data is needed to decide whether inverted CDR3s represent a biological phenomenon or a statistical artifact.

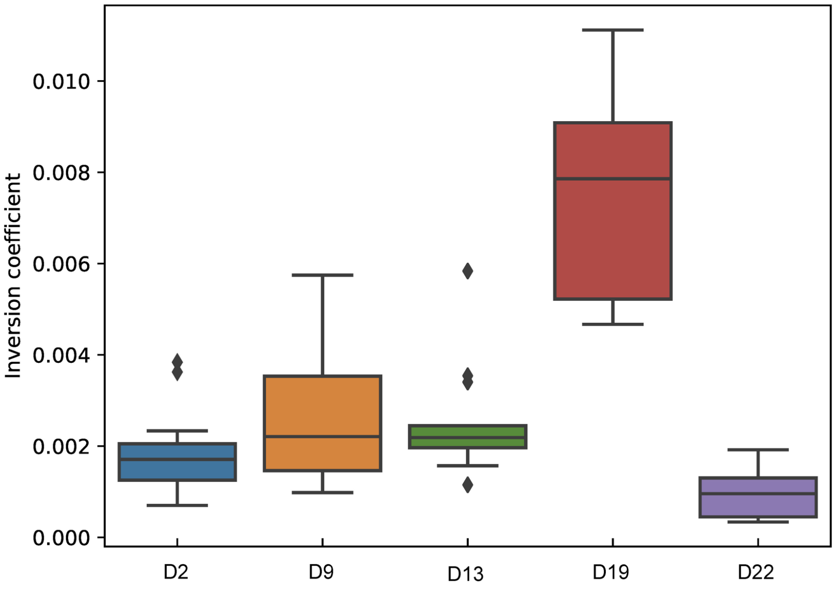

Figure A4. Distribution of the inversion coefficient for D2, D9, D13, D19, and D22 genes.

Supplemental Note: IgScout pseudocode

IgScout takes (i) a set *Strings* representing trimmed CDR3s, (ii) the *k*-mer size, (iii) the information content threshold *IC*, and (iv) the minimum multiplicity *minMultiplicity* of *k*-mers threshold as the input parameters (Figure A5). IgScout selects a most abundant *k*-mer in the *CDR3** dataset (line 5, Figure A5), aligns all CDR3 that contain this *k*-mer (using this *k*-mer as the alignment seed), and constructs the motif logo of the resulting alignment. It uses the **PrefixExtension** and **SuffixExtension** subroutines for extending the selected *k*-mers to the left and to the right and generating the putative D genes. Finally, the algorithm removes the sequences that contain *k*-mers from the identified putative D gene from the set *CDR3** (lines 10–11, Figure A5), finds a most abundant *k*-mer in the resulting dataset (line 5, Figure A5), and iterates. IgScout stops when a most abundant *k*-mer is not a common *k*-mer (line 6, Figure A5).

The choice of default parameters of the algorithm is described in Supplemental Note "IgScout parameters."

01 **IgScout**(*Strings, k, IC, minMultiplicity*)
02 *RemainingStrings* ← *Strings*
03 *D-genes* ← empty set

```
while forever
  D ← a most frequent k-mer in RemainingStrings
  if frequency of D in Strings exceeds minMultiplicity
    D ← PrefixExtension(RemainingStrings, D, k, IC)
    D ← SuffixExtension(RemainingStrings, D, k, IC)
    add string D to the set D-genes
    Strings(D) ←all strings in RemainingStrings containing k-mers from the string D
    remove all strings from Strings(D) from the set RemainingStrings
  else
    return D-genes
```

```
PrefixExtension(Strings, D, k, IC)
prefix ← first k-mer in string D
Strings(prefix) ← all strings in Strings containing prefix
Alignment ← prefix-anchored alignment of all strings in Strings(prefix)
previousColumn ← the column in Alignment preceding the first position of D
if information content of previousColumn exceeds IC
    consensus ← a most frequent nucleotide in previousColumn
    D ← the prefix-extension of the string D by the nucleotide consensus
    PrefixExtension(Strings, D, k, IC)
else
    return D
```

```
SuffixExtension(Strings, D, k, IC)
suffix ← last k-mer in string D
Strings(suffix) ← all strings in Strings containing suffix
Alignment ← suffix-anchored alignment of all strings in Strings(suffix)
nextColumn ← the column in Alignment following the last position of D
if information content of nextColumn exceeds IC
    consensus ← a most frequent nucleotide in nextColumn
    D ← the suffix-extension of the string D by the nucleotide consensus
    SuffixExtension(Strings, D, k, IC)
else
    return D
```

Figure A5. IgScout pseudocode.

Supplemental Note: IgScout parameters

IgScout stops when the most frequent *k*-mer in the remaining CDR3s has abundance below *minMultiplicity=fraction* · |*CDR3**|. We applied IgScout with various values of the parameters *fraction* = {0.0005, 0.001, 0.0015, 0.002, 0.0025, 0.003, 0.0035, 0.004} and *IC* = {30%, 40%, 50%, 60%, 70%, 80%} to 14 HEALTHY immunosequencing datasets. For each launch of IgScout, we computed the following metrics:

- the number of reconstructed D genes (we classify a segment as a reconstructed D gene if it is a substring of this D gene),
- the average number of nucleotides missing at the start/end of the reconstructed D genes,
- the number of novel genes (we classify a segment as novel if it does not represent a substring of a known D gene). Note that novel D genes may represent both allelic variants and false positive inferences.

Figure A6 shows distributions of values of these metrics (averaged over 14 HEALTHY datasets) for each pair of *fraction* and *IC* values and illustrates that fraction = 0.001 and *IC* = 0.5 represent suitable parameters.

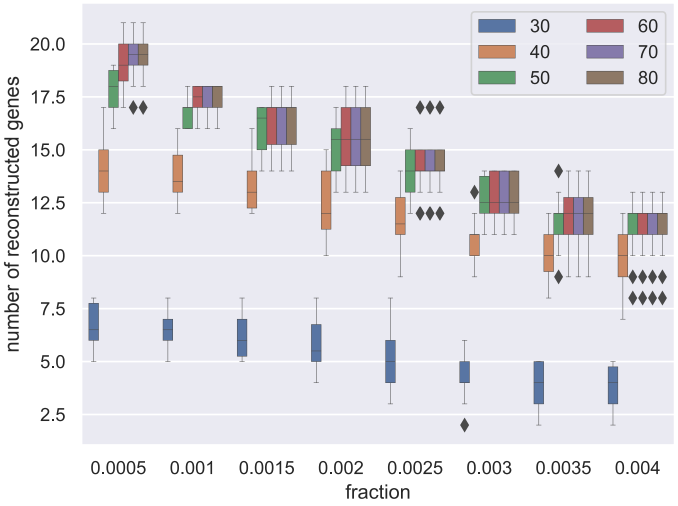

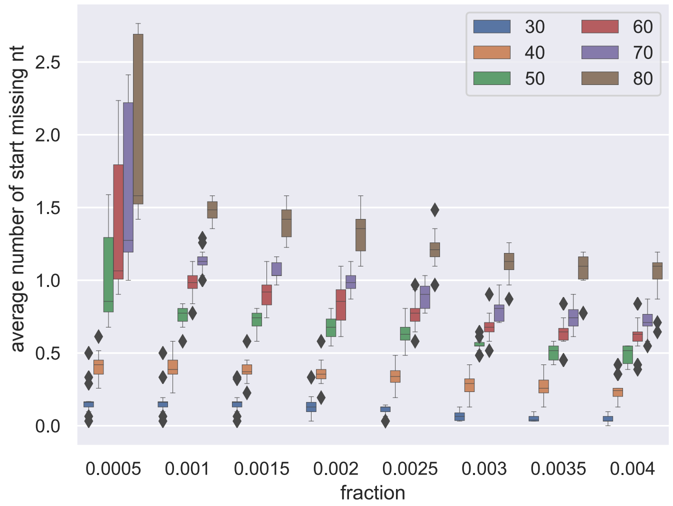

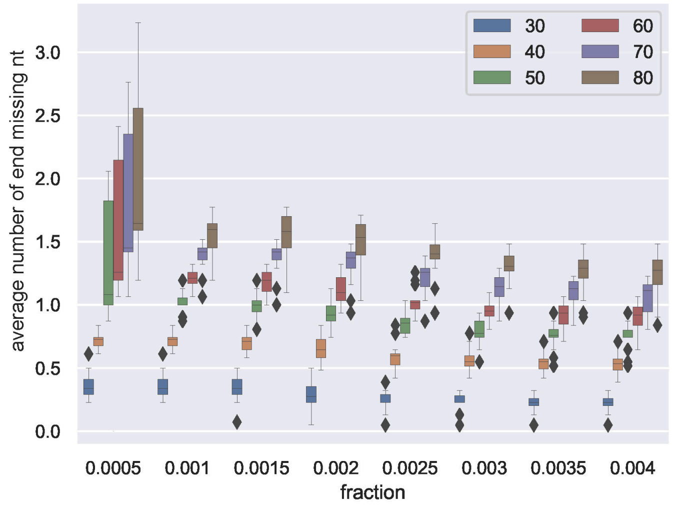

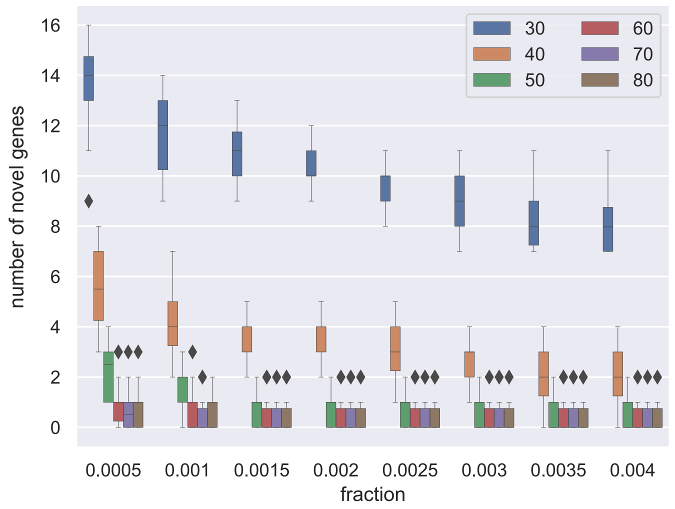

Figure A6. IgScout results for various values of parameters *fraction* and *IC* (the HEALTHY datasets). Colors correspond to various values of *IC* (in percentages). The distribution of the metric values for each pair of *fraction* and *IC* is illustrated as an error bar. The bar shows the quartiles of the distribution, the whiskers demonstrate the rest of the distribution, except for points that are determined as outliers.

Figure A7 shows multiplicities and ranks of known 15-mers after 17 iterations of IgScout (the Set 1 dataset). IgScout stops before reconstructing the D7 gene because its most abundant 15-mer occurs in less than *fraction*=0.1% of strings in the *CDR3** dataset. Although decreasing the *fraction* threshold would lead to reconstructing additional D genes, it may also add false positive reconstructions. Figure A8 shows abundances of common 15-mers in the set of CDR3s that remain after IgScout completed its work.

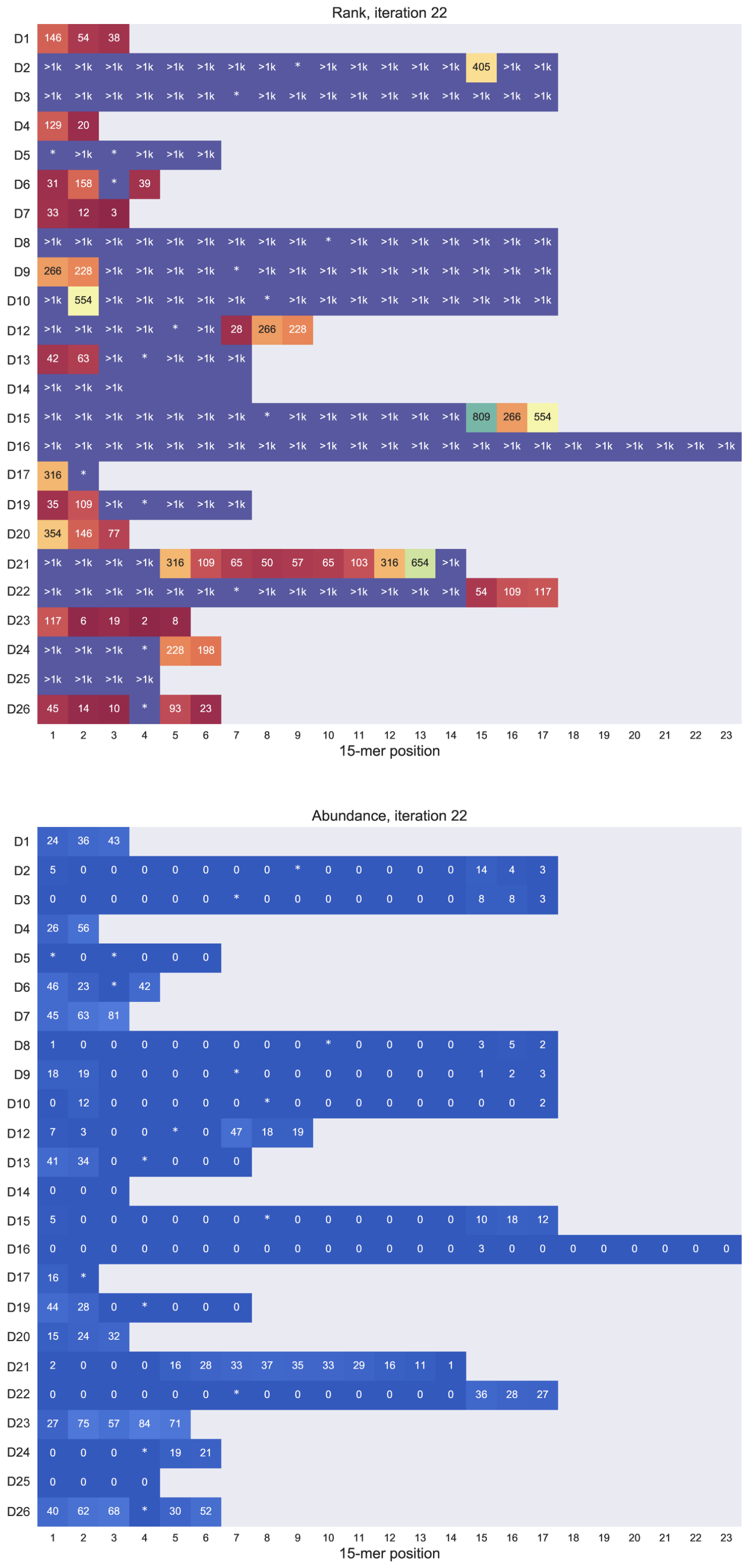

Figure A7. Ranks (top) and abundances (bottom) of 15-mers from known D genes constructed for the CDR3s remaining after IgScout completed its work (for the Set 1 dataset). 15-mers used for inference of D genes are marked with “*”.

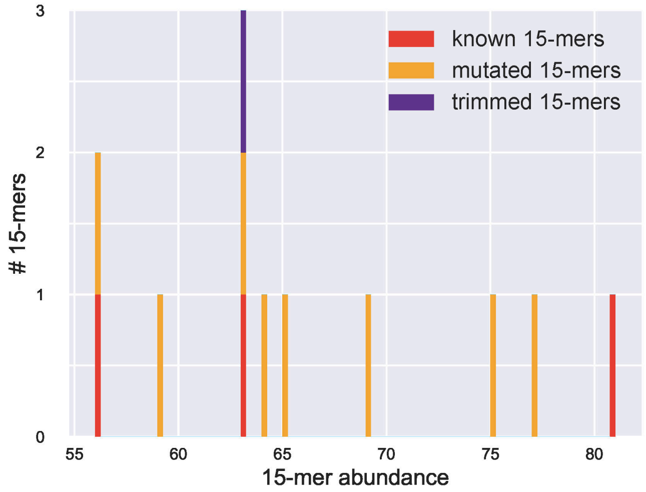

Figure A8. Abundances of all 12 common 15-mers in the *CDR3 set after 17 iterations of IgScout.** We removed CDR3s participating in the inference of novel D segments during the first 17 steps of the IgScout algorithm and analyzed all common 15-mers in the remaining 55,514 CDR3s. These 12 common 15-mers have abundances varying from 56 to 81. The *y*–axis represents the number of common 15-mers with the given abundance. Red, orange, and violet bars represent the number of common 15-mers with given abundance among common 15-mers. There exist 3 known (red bars), 8 mutated (orange bars) and 1 trimmed (violet bars) 15-mers. There are no foreign 15-mers among common 15-mers after the IgScout run. The three known 15-mers belong to genes D7 and D15.

Supplemental Note: Simulating CDR3 datasets

To generate simulated immunosequencing datasets, we used the IgSimulator tool (Safonova et al., 2015) with the set of 25 human D genes (D1–D27). For the sake of simplicity, we first assumed that all 25 D genes participate in VDJ recombination with the same probability 0.04 (uniform distribution of abundances) and later analyzed a non-uniform distribution of abundances. As a variable parameter of the simulation, we used the maximal length of the exonuclease removal (*ERmax*). To simulate a substring of a D gene in the VDJ recombination, we randomly selected integers *ERstart* and *ERend* (uniformly distributed between 0 to *ERmax*), cropped the sequence of a D gene by *ERstart* nucleotides from the start and *ERend* nucleotides from the end, and added random insertions (with the length uniformly distributed from 0 to 10 nucleotides) on both ends. We varied *ERmax* from 1 to 10 (according to Ralph and Matsen, 2016, *ERmax* typically does not exceed 10 nucleotides for all D genes).

IgSimulator simulates SHMs as low-frequency random mutations. Since such SHMs are unlikely to change the IgScout results (most mutated D segments in CDR3 will be simply excluded from analysis since they do not preserve *k*-mers), we decided not to simulate clonal lineages with abundant SHMs. Indeed, a new Supplemental Note "How IgScout results are affected by the number of consensus CDR3s and cell types?" demonstrates that IgScout shows better results on datasets with high diversity of VDJ recombination (datasets from PBMC / naïve / memory B cells) compared to highly mutated datasets with low diversity of VDJ recombination (e.g., datasets from specific plasma B cells).

We simulated 10 datasets with 100,000 CDR3s each (*ERmax* varies from 1 to 10) for and applied IgScout with default parameters (k = 15, *fraction* = 0.001, *IC* = 0.5) to each of them. We assume that IgScout reconstructs a D gene if it reports its unique substring (i.e., a substring that does not appear in other D genes). Below we discuss how the length of the reconstructed D genes influences downstream applications of IgScout.

To analyze how our ability to reconstruct low-usage D genes is affected by non-uniform distribution of usage of D gene, we have simulated a repertoire with a high usage of a single gene (we selected the longest D gene D16) and low usages of all other genes (Table A8).

D gene	Usage	D gene	Usage
D1	0.001	D15	0.0205
D2	0.0025	D16	0.5620
D3	0.0040	D17	0.0220
D4	0.0055	D19	0.0235
D5	0.0070	D20	0.0250
D6	0.0085	D21	0.0265
D7	0.0100	D22	0.0280
D8	0.0115	D23	0.0295
D9	0.0130	D24	0.0310
D10	0.0145	D25	0.0325
D12	0.0160	D26	0.0340
D13	0.0175	D27	0.0355
D14	0.0190		

Table A8. Simulating CDR3s with non-uniform usage of D genes. Abundance of a D gene shows the fraction of CDR3s in the simulated repertoire formed by this D gene. We arbitrarily assigned abundances varying from 0.001 to 0.025 (with step 0.0015) to all genes except for D16. The sum of these abundances is 0.438 and abundance of D16 was set to 1 – 0.438 = 0.562.

Supplemental Note: Benchmarking IgScout on simulated immunosequencing datasets

We applied IgScout to simulated CDR3 datasets with uniform and non-uniform usage of D genes (see Supplemental Note "Simulating CDR3 datasets").

IgScout results in the case of the uniform distribution of usage of D genes. IgScout reconstructed 24 out of 25 D genes for all values of *ERmax* (short 11-nucleotide long gene D27 cannot be detected with k = 15). On average, IgScout misses one nucleotide at the start of D gene and one nucleotide at the end D gene for all values of *ERmax*. In all simulations, IgScout returned erroneous D genes only for unrealistically small values *ERmax* = 1 and 2. Our simulation suggests that IgScout would likely reconstruct all D genes (except for a short D27) if their abundances were uniformly distributed.

IgScout results in the case of a non-uniform distribution of usage of D genes. Figure A9 shows that IgScout reconstructs long D genes (> 20 nt) even if they are presented in less than 1% of CDR3s. IgScout missed short D genes (< 20 nt) when their abundance falls below 2.5% (D1, D4, D5, D6, D7, D14, D17, D20). IgScout also missed D27 because it is shorter than the default value of k=15.

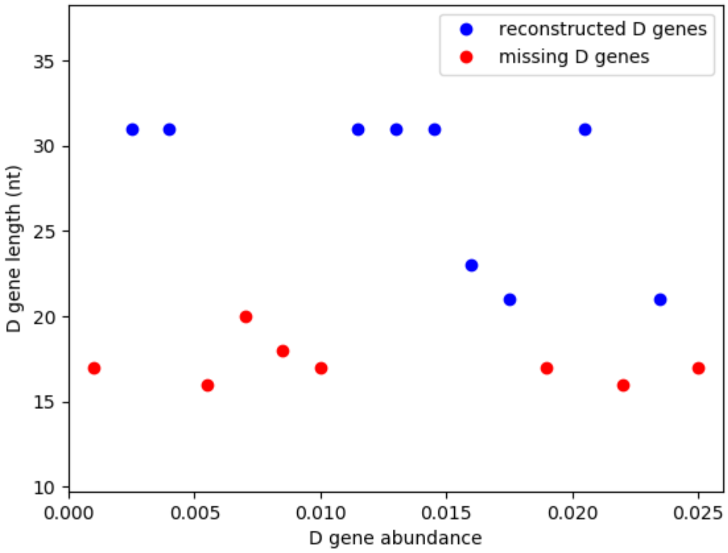

Figure A9. Performance of IgScout on simulated CDR3s with non-uniform usage of D genes. D genes reconstructed (missing) by IgScout are shown by blue (red) dots. Usages of D genes are shown in Table A8.

Supplemental Note: How trimmed (rather than complete) D genes affect the downstream analysis of immunosequencing datasets

Two modes of IgScout applications. IgScout can be applied for:

- inference of novel variants of known D genes (for species with known germline genes) and further population analysis of antibody repertoires,
- inference of D genes (for species with unknown germline genes) and further VDJ classification (i.e., finding V, D, and J genes explaining the observed VDJ recombination).

The first *reference-based* mode does not require inference of full-length D genes because they can be reconstructed from the trimmed D genes by aligning against the known variants of D genes (IgScout has a reference-based mode "*--d-genes*" that compares the reconstructed D genes with known ones). However, the negative impact of the reduced lengths of the inferred D genes on the quality of the D gene classification is unclear. Below we show that this impact is very small.

Defining a match between a CDR3 and a D gene. Existing VDJ classification tools search for a match between a CDR3 and a D gene with the score exceeding a threshold *L*. To estimate the accuracy of the D gene classification, we used the datasets simulated with *ERmax* = 10 (that we refer to as the SIMULATED dataset) with uniform abundances of D genes (see Supplemental Notes "Simulating CDR3 datasets").

We analyzed a simple scoring based a longest match between each CDR3 from the SIMULATED dataset and each D gene from a database. We say that a CDR3 is *generated* by a specific D gene if this D gene results in a longest match with this CDR (over all D genes from the database). If several D genes provide the longest matches, we say that all of them generated a given CDR3. Since we did not simulate SHMs, we compute only the exact matches and thus produce more accurate results for the SIMULATED dataset compared to IgBlast (Ye et al., 2013) that allows mismatches and indels. Note that an algorithm for D gene classification that takes into account mismatches and indels might generate less accurate results than an algorithm based on exact matches since it may be confused by highly similar D genes (e.g., it can extend an exact match by mismatches and report an incorrect D gene).

False positive and false negative CDR3 classifications. Given a simulated CDR3 (referred to as *CDR3*), we refer to the D gene it originated from as *D(CDR3)* and to D genes with a longest match against this CDR3 as *D*=D*(CDR3).* We refer to this match as *match(CDR3,D*).* We further check if the found longest match exceeds the length threshold *L* and classify *CDR3* as follows:

1. If |*match(CDR3,D*)*| ≥ *L* and *match(CDR3,D*)* represents a unique substring of *D(CDR3),* we classify *CDR3* as a true positive (TP) and as false negative (FN) otherwise.
2. If |*match(CDR3,D*)*| < *L,* we classify *CDR3* as a true negative (TN), and a false positive (FP) otherwise.

Using the classification of all CDRs, we compute the *sensitivity* as #TP / (#TP + #FN) and the *specificity* as #TN / (#TN + # FP) for values of the length threshold *L* varying from 5 to 15 nucleotides.

We applied this procedure to the following three datasets of D genes to study the negative effects of trimmed D genes as compared to the full-length D genes:

- 25 complete human D genes (referred to as the COMPLETE database)
- 24 *trimmed* human D genes inferred by IgScout for the SIMULATED dataset (referred to as the TRIMMED database)
- 17 *highly trimmed* D genes represented by the most abundant 15-mers selected by IgScout for inference of these D genes in the HEALTHY dataset (referred to as $TRIMMED^+$ database)

Sensitivity and specificity of the D gene classification. Figure A10 shows the sensitivity and specificity of the classification of an arbitrarily selected single D gene (D13) in all CDR3s generated by this D gene from the SIMULATED dataset. As Figure A10 illustrates, the sensitivity and specificity of the complete and trimmed D genes are very similar. On the other hand, the low sensitivity of the D gene classification using 15-mers in the $TRIMMED^*$ dataset demonstrates that the extension of abundant 15-mers by IgScout is an important step that significantly improves the D gene classification.

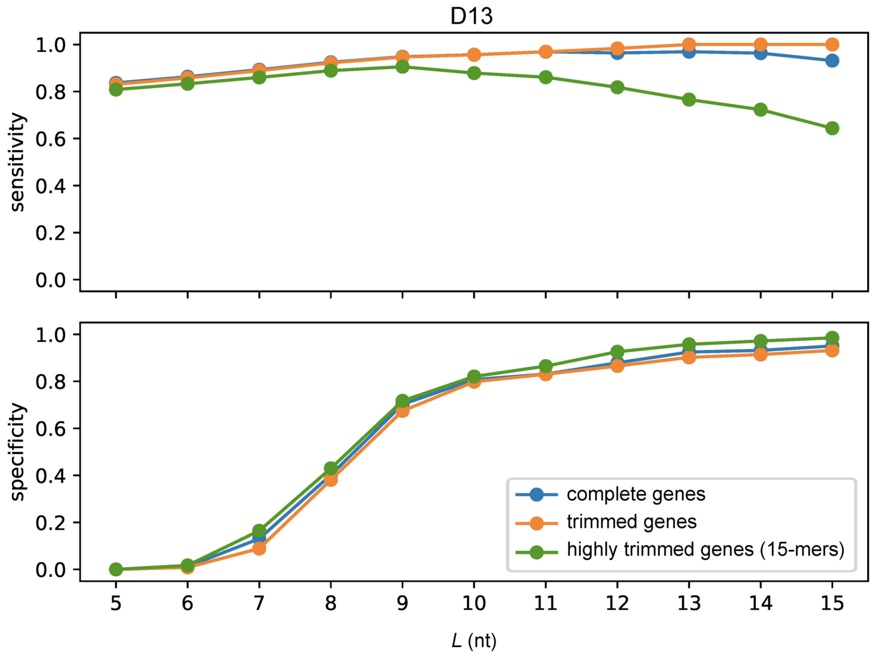

Figure A10. Sensitivity and specificity of classifying the D13 gene in the CDR3s from the SIMULATED dataset. We used the COMPLETE (blue), TRIMMED (orange), and $TRIMMED^+$ (green) datasets of D genes for classifying CDR3s. The length threshold *L* varied from 5 to 15 nucleotides.

Figure A11 illustrates that the sensitivity/specificity of the D gene classification of complete D genes and trimmed D genes are nearly identical (for all values of L). Thus, trimming 1-2 nucleotides at the start/end of D genes hardly affects the accuracy of the D gene classification.

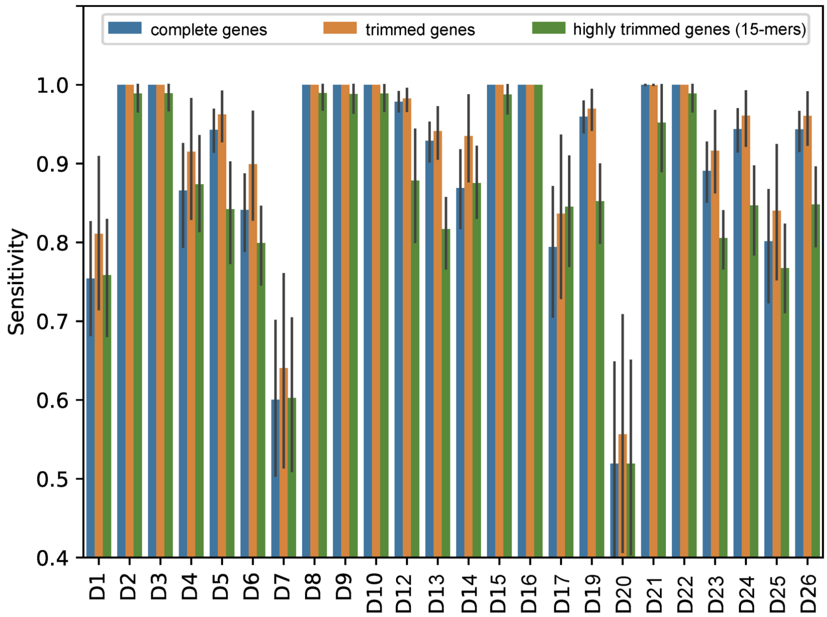

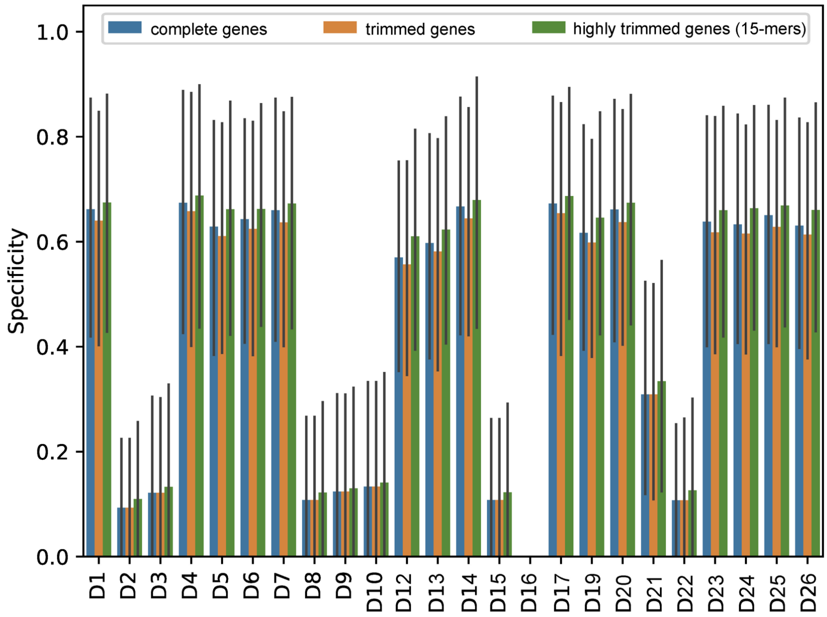

Figure A11. Sensitivity (top) and specificity (bottom) of classifying 24 inferred D genes in the SIMULATED dataset. Each bar represents the sensitivity (specificity) for various values of the length threshold L. Height of a bar shows the average value of sensitivity (specificity). Error bars shown by black lines correspond to the minimal and maximal values of sensitivity (specificity).

Supplemental Note: Reconstructing variants of human D genes

Figure A12 presents information about reconstructed D genes across the HEALTHY datasets.

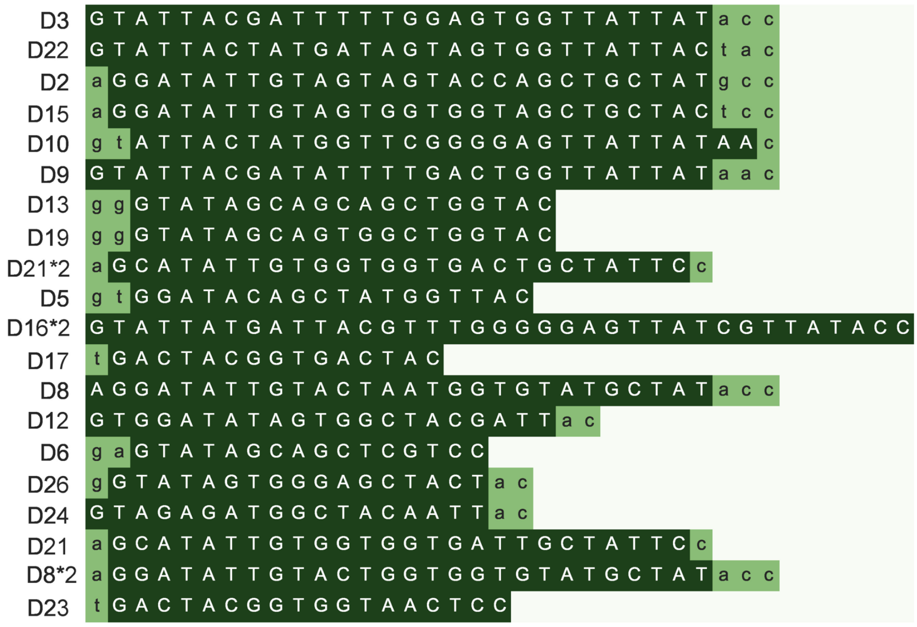

Figure A12. Information about D genes reconstructed by IgScout across all HEALTHY datasets. Position in a D gene is colored in dark green if it was reconstructed in at least one of the HEALTHY datasets. Positions in D genes that were not reconstructed in all datasets are shown in light green. Ordering of rows reflects the order in which IgScout discovers various D genes, e.g., D3 appears in the first row because it was reconstructed at the first step of IgScout in 7 out of 14 datasets, D22 appears in the second row because it was reconstructed at the first step of IgScout in 6 out of 14 datasets. If IgScout took n steps to analyze the i-th dataset and reconstructed a gene D at the j-step we assign $index(D,i)=j$ and assign $index(D,i)=n+1$ if IgScout failed to reconstruct the gene D in the i-th dataset. All D genes are arranged from top to bottom in the increasing order of the average values of their indices across all fourteen datasets. Genes D1, D4, D7, D14, D20, D25, and D27 are not shown since they were not discovered in any of the HEALTHY datasets.

Table A9 illustrates that IgScout finds novel variants D10$^+$ (D16$^+$) in 50 (46) datasets from 600 PROJECTS10 immunosequencing datasets. It also found novel D10^{++} and D16^{++} in two datasets from the PRJNA308566 project and one dataset from the PRJNA308641 project, respectively (Figure A13). All variants of the D16 gene (D16, D16*2, D16$^+$, and D16^{++}) differ from each other in two positions that can be described as 0-0, 0-1, 1-1, and 1-0 haplotypes for D16, D16*2, D16$^+$, and D16^{++}, respectively.

NCBI project	# datasets	# datasets supporting D10+	# datasets supporting D16+
PRJEB18926	24	8	8
PRJNA396773	13	13	13
PRJNA308641	107	9	8
PRJNA324093	95	–	3
PRJNA248475	32	–	–
PRJNA308566	142	6	3
PRJNA355402	93	1	1
PRJNA393446	42	8	7
PRJNA349143	24	–	–
PRJNA430091	28	5	3

Table A9. Information about immunosequencing datasets in the PROJECTS10 collection supporting the novel D10$^+$ and D16$^+$ variants. The “# datasets” column shows the total number of datasets in each project.

```
D10+   GTATTACTATGGTTCAGGGAGTTATTATAAC   D16+   GTATTATGATTACATTTGGGGGAGTTATCGTTATACC
D10++  GTATTACTATGGGTCGGGGAGTTATTATAAC   D16++  GTATTATGATTACATTTGGGGGAGTTATGCTTATACC
D10    GTATTACTATGGTTCGGGGAGTTATTATAAC   D16    GTATTATGATTACGTTTGGGGGAGTTATGCTTATACC
D10*2  GTATTACTATGTT-CGGGGAGTTATTATAAC   D16*2  GTATTATGATTACGTTTGGGGGAGTTATCGTTATACC
```

Figure A13. Novel variants of D10 and D16 genes. The D10^{++} variant was inferred from the datasets SRR3099127 and SRR3099139 (project PRJNA308566) corresponding to the same individual. The D16^{++} variant was inferred from the SRR3099414 dataset (project PRJNA308641).

Supplemental Note: Summary of IgScout results across diverse immunosequencing datasets

We applied IgScout to 361 Rep-seq datasets from ten independent immunosequencing projects corresponding to diverse immunogenomics studies (Table A9). Figure A14 shows the sets of reconstructed D genes for each dataset and illustrates that 20 D genes were reconstructed across all datasets. In addition to 18 D genes inferred from the HEALTHY datasets, IgScout reconstructed D4 (in 3 datasets) and D7 (in 5 datasets). Five D genes (D1, D14, D20, D25, and D27) are missing in all analyzed datasets. These five genes are also reported as missing in multiple studies on analyzing the usage of D genes: Souto-Carneiro et al., 2005, Briney et al., 2012, Elhanati et al., 2015, and Kidd et al., 2016. For example, Briney et al., 2012 reported three D genes (D14, D20, D27) as not contributing to VDJ recombination in all their datasets, while Elhanati et al., 2015 reported the same three genes as well as six other D genes as (D4, D5, D7, D12, D24, D26) as missing in their datasets.

As Figure A14 illustrates, even the most abundant D genes are missing in some datasets, e.g., D20 was identified in all HEALTHY datasets but was not identified in 10% of the 361 datasets. These datasets likely represent repertoires where a single D gene with a very high usage overpowers all others D genes because of a clonal selection (e.g., in flu vaccination study PRJNA324093 and in hepatitis vaccination study PRJNA308566). Supplemental Note "How IgScout results are affected by the number of consensus CDR3s and cell types" discusses IgScout performance on such datasets.

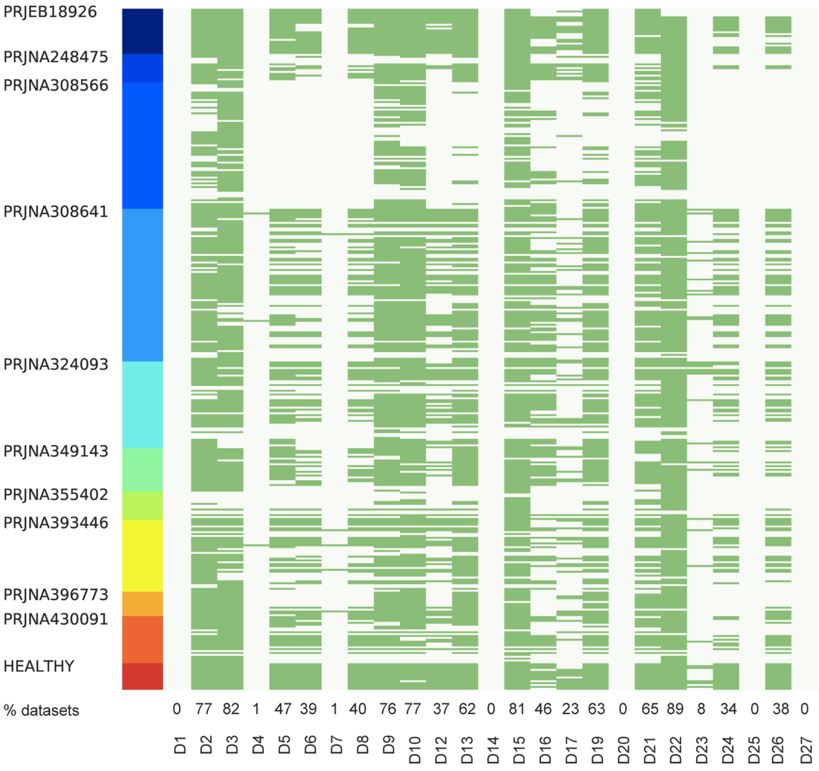

Figure A14. Human D genes that were reconstructed (green cells) and missed (white cells) by IgScout in 361 immunosequencing datasets from ten NCBI projects (Table A9) and the HEALTHY datasets.

Datasets corresponding to the same NCBI project are grouped together and shown by a colored bar on the left. 14 HEALTHY datasets are shown at the bottom of the table. The percentage of the datasets supporting inference of each of 25 human D genes is shown in the row "% datasets".

Supplemental Note: How IgScout results are affected by the number of consensus CDR3s and cell types?

To evaluate how IgScout results are affected by the number of consensus CDR3s and cell types, we analyzed two NCBI immunosequencing projects containing 242 datasets with B cells sorted by their type and antigen specificity (Table A10).

NCBI project	# datasets	description	analyzed B cells	analyzed tissues
PRJNA308566	142	Hepatitis vaccination study	PBMC, HBsAg+ cells, HLA-DR+ plasma cells	blood
PRJNA324093	100	Flu vaccination study	PBMC, memory, naïve, HA+ ASCs, HA– ASCs	blood

Table A10. Information about the PRJNA308566 and PRJNA324093 projects with 242 immunosequencing datasets. HBsAg+ / HLA-DR+ / HA+ refer to cells with positive response to HBsAg, HLA-DR, and hemagglutinin, respectively. HA– refers to cells with negative response to hemagglutinin. ASC refers to antibody secreting cells.

Some of the datasets from PRJNA308566 and PRJNA308566 projects are characterized by a low number of consensus CDR3s (< 5000). Such low-diversity datasets likely correspond to situations when one clonal lineage (or a few clonal lineages) has an extremely high abundance as compared to all other clonal lineages. Since IgScout was not designed to analyze such datasets, it did not reconstruct any D genes in 75 datasets from PRJNA308566 and 49 datasets from PRJNA308566. Figure A15 presents the summary of IgScout results for all other datasets.

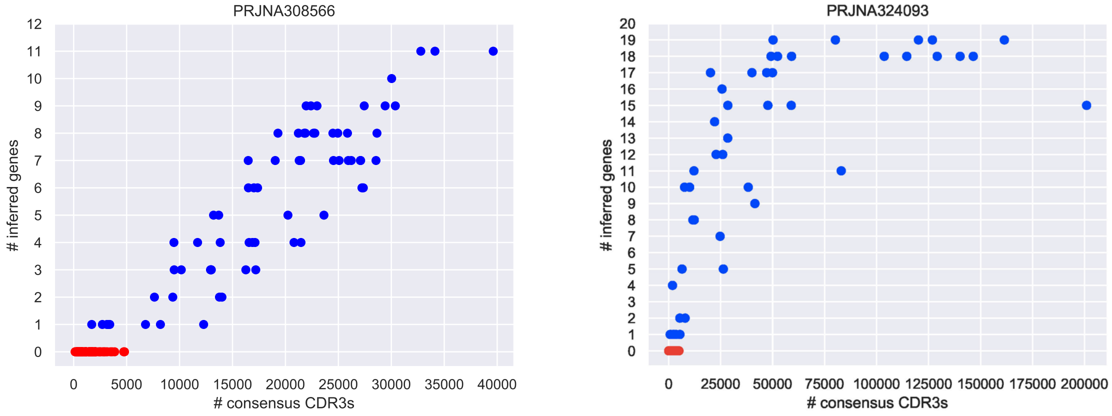

Figure A15. The number of inferred D genes vs the number of consensus CDR3s for datasets from the PRJNA308566 (left) and PRJNA324093 (right) projects. Each dataset corresponds to a single dot. Datasets without inferred D genes are shown as red dots.

We also analyzed how the type of cells in a dataset affects the IgScout results. Figure A16 shows that IgScout results depend mainly on the number of consensus CDR3s in a dataset rather than the type of B cells in this dataset. As Figure A16 illustrates, IgScout reconstructed D genes even from datasets corresponding to highly specific B cells (e.g., HBsAg+ or ASC-HA+). However, it is important to take into account that the number of consensus CDR3s is correlated with the number of different VDJ recombinations in a dataset. Thus, a small number of VDJ recombinations, occurring in datasets with highly specific B cells, may lead to a small number of inferred D genes.

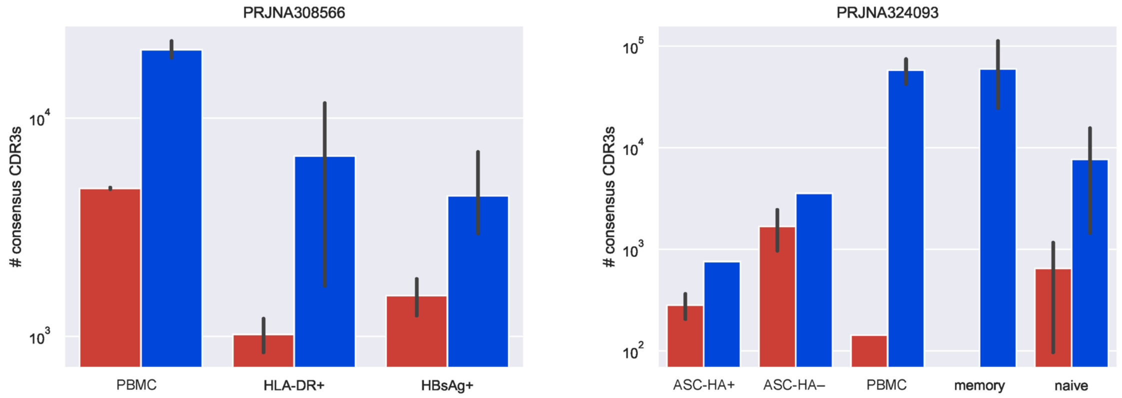

Figure A16. IgScout results on datasets corresponding to various types of B cells in the PRJNA308566 (left) and PRJNA324093 (right) projects. Each bar represents datasets corresponding to the same type of B cells. The height of a bar shows the average number of consensus CDR3s in these datasets (in the logarithmic scale), the error bars show the distribution of the numbers of consensus CDR3s. Red bars correspond to datasets where IgScout did not infer any D genes and blue bars correspond to datasets where IgScout inferred some D genes.

Supplemental Note: Reconstructing camel D genes

IgScout reconstructed four D genes in the case of the Camel 1VH dataset that we refer to as D1, D2, D3, and D4 (Figure A17). IgScout reconstructed the same or very similar putative D genes in all camel datasets (Table A11) but missed D4 in datasets 2VHH, 3VH, and 3VHH (all camel D genes are shared between the VH and VHH datasets). Table A11 shows abundances of common 15-mers in the Camel 1VH dataset before and after the IgScout run.

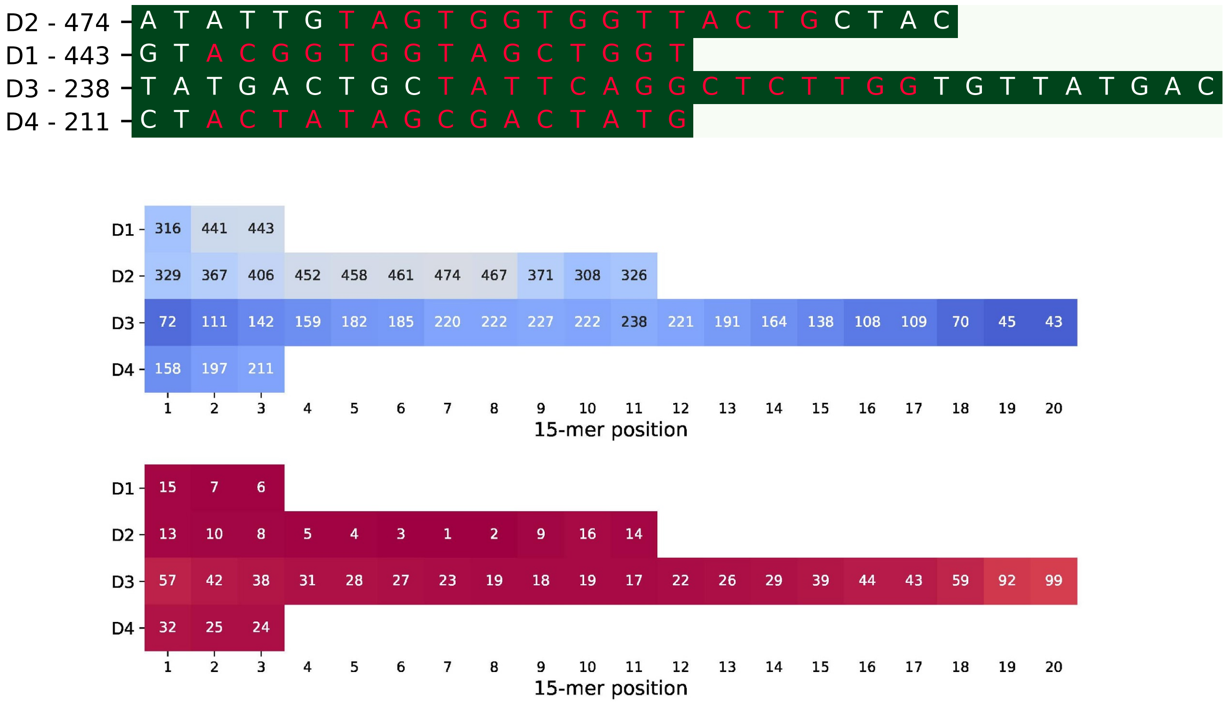

Figure A17. Results of the IgScout algorithm on the Camel 1VH dataset. Four inferred D genes in the Camel 1VH dataset (top), abundances (middle) and ranks (bottom) of 15-mers from the inferred camel D genes. Details of this visualization are described in the legends for Figure 2 and Figure A3.

D1		D2	
Camel 1VH	GTACGGTGGTAGCTGGT	Camel 1VH	ATATTGTAGTGGTGGTTACTGCTAC

Camel 1VHH GTACGGTGGTAGCTGGT	Camel 1VHH ATATTGTAGTGGTGGTTACTGCTAC
Camel 2VH GTACGGTGGTAGCTGGT	Camel 2VH CGCATACTATAGTGGTGGTTACTACTAC
Camel 2VHH GTACGGTGGTAGCTGGT	Camel 2VHH ATATTGTAGTGGTGGTTACTGC---
Camel 3VH GTACGGTGGTAGCTGGT	Camel 3VH ATATTGTAGTGGTGGTTACTGCTAC
Camel 3VHH GTACGGTGGTAGCTGGT	Camel 3VHH CATATTGTAGTGGTGGTTACTGC---
D3	D4
**Camel 1VH TATGACTGCTATTCAGGCTCTTGGTGTTATGAC**	**Camel 1VH CTACTATAGCGACTATG**
Camel 1VHH GTATGACTGCTATTCAGGCTCTTGGTGTTATGAC	Camel 1VHH CTACTATAGCGACTATG
Camel 2VH GTATGACTACTGTTCAGGCTCTTGGTGTTATG--	Camel 2VH CTACTATAACGAATATGAC
Camel 2VHH GTATGACTGCTATTCAGGCTCTTGGT--------	
Camel 3VH TATGACTGCTATTCAGGCTCTTGGTGTTATG--	
Camel 3VHH -ATGACTGCTATTCAGGCTCTTGGTG-------	

Table A11. Constructing four putative camel D genes. Strings inferred for the Camel 1VH are shown in bold. Differences between the strings inferred for the Camel 1VH dataset and strings inferred from other datasets are shown in red.

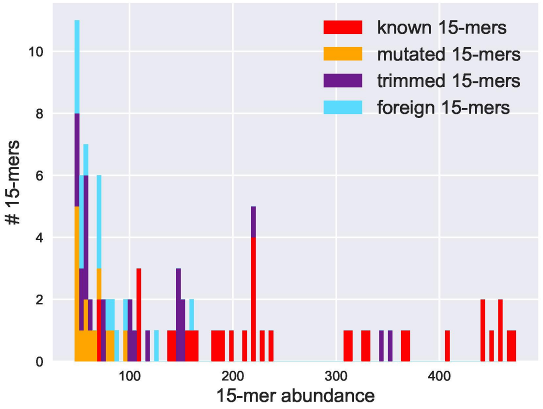

Figure A18. Abundances of all common 15-mers in the Camel 1VH dataset. 89 common 15-mers in the Camel 1VH dataset have abundances varying from 48 to 474. The *y*–axis represents the number of common 15-mers with the given abundance. Red, yellow, and violet bars represent the number of common 15-mers with given abundance among known, mutated, and trimmed 15-mers. There exist 35 known (red bars), 14 mutated (orange bars), 24 trimmed (violet bars), and 16 foreign (blue bars) common 15-mers.

Table A12 provides information about the fraction of traceable and tandem CDR3s in various camel datasets. Figure A19 provides information about the usage of four inferred camel D genes. Although all four D genes occur in both VH and VHH antibodies, their usage varies depending on the antibody type. Conrath et al., 2003 hypothesized that the same D and J genes are used for forming both the VH and VHH camel antibodies. If this hypothesis is correct, then the variations in the usage of D genes in the VH and VHH antibodies are most likely caused by differences between the RSSs in the V genes in the VH and VHH loci.

dataset	traceable CDR3s		tandem CDR3s		non-traceable CDR3s	
	# (%)	avg. length	# (%)	avg. length	# (%)	avg. length
Camel 1VH	10,224 (22%)	55	176 (0.4%)	69	35,926 (77.6%)	54
Camel 1VHH	8443 (21.2%)	60	222 (0.6%)	73	31,036 (78.2%)	60
Camel 2VH	12,158 (21%)	54	183 (0.3%)	70	45,777 (78.7%)	53
Camel 2VHH	17,356 (23.3%)	56	1292 (1.7%)	61	56,198 (76%)	54
Camel 3VH	17,289 (21.8%)	51	1124 (1.4%)	59	60,969 (76.8%)	46
Camel 3VHH	13546 (23%)	56	1068 (1.8%)	62	44708 (75.2%)	53

Table A12. Classification of CDR3s across six camel immunosequencing datasets. Since only a small percentage of CDR3s in camel immunosequencing datasets contains 15-mers from the inferred camel D genes, we defined a traceable CDR3 as a CDR3 that include 15-mers with up to two mutations from 15-mers from the inferred camel D genes.

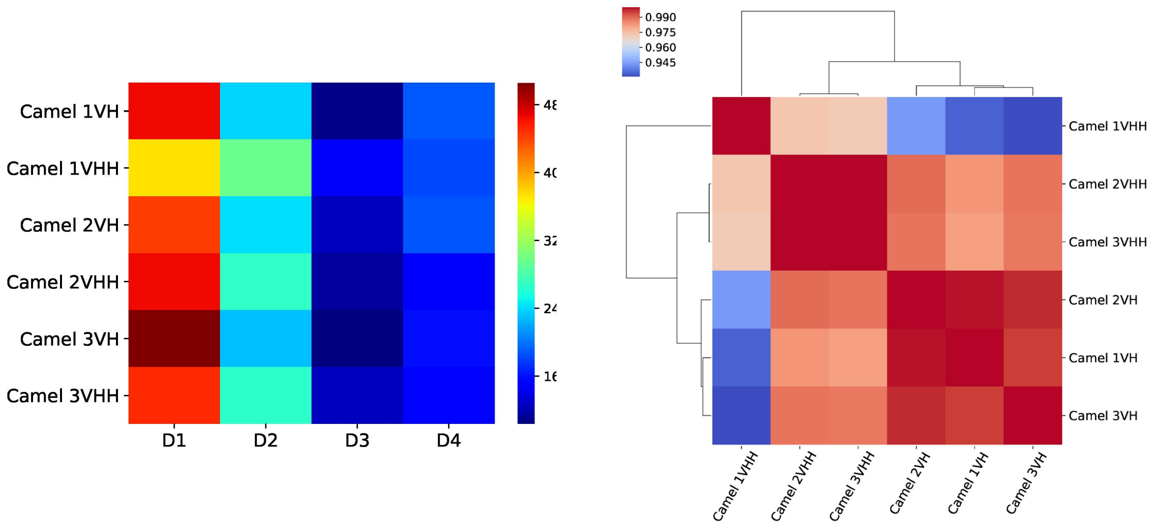

Figure A19. Usage of four camel D genes across six camel datasets (left) and the similarity matrix of camel datasets constructed based on usages of their D genes (right).

Figure A20 presents comparison between the four camel D genes and eight alpaca D genes listed in the IMGT database (camel and alpaca split ≈16 million years ago). For each camel D genes, there exists a similar alpaca D gene with percent identity varying from 82% to 94%.

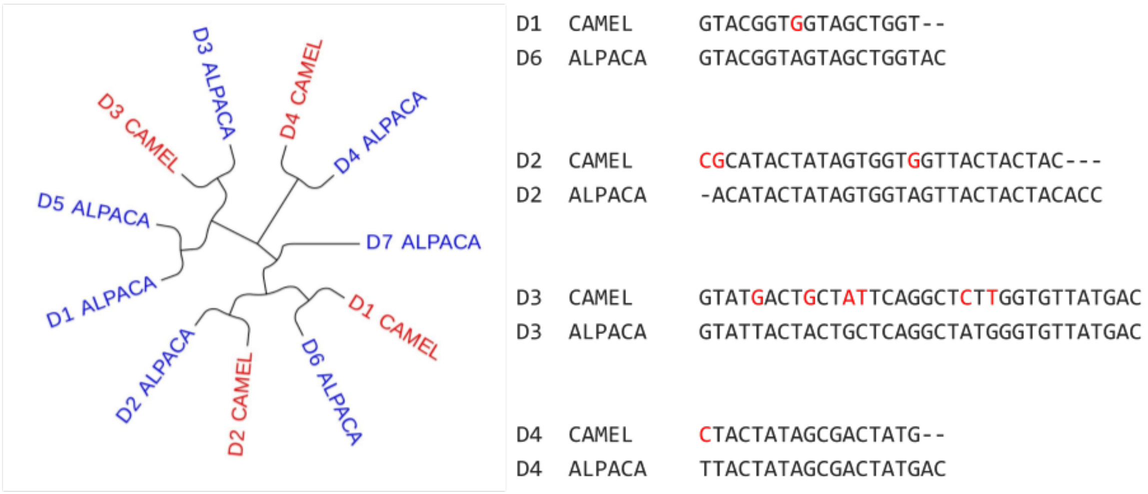

Figure A20. **Comparison of four camel D genes with eight alpaca D genes.** (Left) Phylogenetic tree for combined camel (blue) and alpaca (red) D genes. (Right) Alignment of four pairs of similar camel and alpaca genes. Differences between camel and alpaca D genes are shown in red.

Supplemental Note: Traceable CDR3s

Figure A21 illustrates the usage of all human D genes across all HEALTHY datasets. Table A13 illustrates that the percentage of traceable (tandem) CDR3s varies from 43% to 55% (0.1 – 0.2%) across all HEALTHY datasets. The average length of traceable, tandem, and non-traceable CDR3s is 53, 71, and 40 nucleotides, respectively.

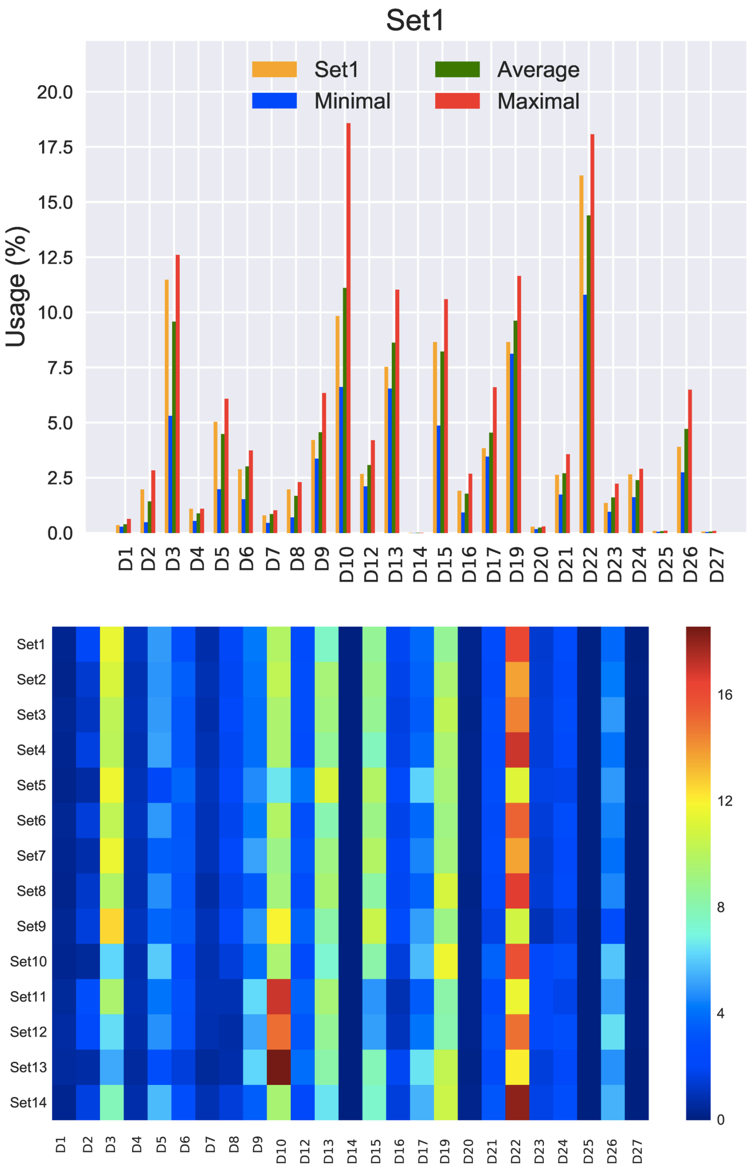

Figure A21. Usage of human D genes across all HEALTHY datasets. (Top) Usage of human D genes in the Set 1 dataset (yellow bars) compared to the minimal (blue bars), average (green bars), and maximal (red bars) usage of D genes across all HEALTHY datasets. (Bottom) Each cell shows the percentage of CDR3s formed by the corresponding D gene (x-axis) in the corresponding dataset (y-axis).

dataset	traceable CDR3s		tandem CDR3s		non-traceable CDR3s	
	# (%)	avg. length	# (%)	avg. length	# (%)	avg. length
Set 1	37938 (46%)	54	114 (0.1%)	72	44528 (54%)	49
Set 2	34768 (46%)	54	161 (0.2%)	71	40470 (54%)	48
Set 3	14492 (44%)	53	45 (0.1%)	70	18552 (56%)	48
Set 4	47764 (50%)	53	159 (0.1%)	73	47296 (60%)	48
Set 5	54997 (46%)	53	145 (0.1%)	68	63600 (54%)	47
Set 6	34900 (46%)	54	122 (0.1%)	71	40886 (54%)	48
Set 7	54180 (43%)	53	123 (0.1%)	70	70435 (56%)	47
Set 8	31072 (46%)	53	94 (0.1%)	70	36157 (54%)	48
Set 9	68664 (52%)	54	263 (0.2%)	72	63563 (48%)	49
Set 10	56873 (55%)	52	127 (0.1%)	69	45700 (44%)	47
Set 11	38381 (45%)	53	87 (0.1%)	70	45916 (54%)	48
Set 12	54674 (48%)	51	110 (0.1%)	70	60195 (52%)	46
Set 13	64696 (50%)	52	109 (0.1%)	70	65721 (50%)	46
Set 14	63610 (53%)	52	151 (0.1%)	72	55933 (47%)	47

Table A13. Information about traceable, tandem, and non-traceable CDR3s across all HEALTHY datasets.

Supplemental Note: D gene classification by IgScout and IgBlast

We compared the D gene classification results generated by IgScout and IgBlast. Since IgBlast computes alignments for full-length immunoglobulin sequences, we analyzed raw reads for the Set1 dataset. 1,414,503 out of 1,611,497 reads (87%) were classified as CDR3-containing reads by both IgBlast and DiversityAnalyzer.

We classify a CDR3 as *non-traceable* if IgBlast reports several best D hits with the same alignment score. 550,514 out of 1,414,503 CDR3s (39%) are non-traceable. We also discarded 287,881 CDR3s (20%) because the D hits found by IgBlast are short and thus unreliable (shorter than 11 nt). For the remaining 576,108 CDR3s with putative D hits, we compared hits reported by IgBlast with hits reported by IgScout. For 504,028 out of 576,108 CDR3s (87%), IgBlast and IgScout report identical D hits. For 4613 CDR3s, IgScout reported tandem D genes (1%). The vast majority of the remaining 12% of CDR3s (where IgBlast and IgScout disagreed) correspond to similar D genes (e.g., the 31-nucleotide long D22 and D9 that share a 7-mer and a 9-mer). In this case, different scoring schemes produce slightly different results and it is not clear how to select the best one.

Supplemental Note: Analysis of tandem CDR3s

IgScout identified 1900 tandem CDR3s in fourteen immunosequencing datasets corresponding to 225 distinct pairs of D genes (*D-pairs*). For each D-pair, we define the *D-pair abundance* as the number of tandem CDR3s formed by D genes in the pair and classify *abundant D-pairs* as the D-pairs with abundances exceeding 1% of the number of all tandem CDR3s. 27 abundant D-pairs include 15 D genes and form 916 out of 1900 tandem CDR3. Figure A22 presents a graph with 16 vertices corresponding to D genes participating in abundant D-pairs (gene D5 corresponds to two vertices since it occurs twice in the IGH locus) and 27 edges (corresponding to abundant D-pairs). This graph turned out to be an acyclic directed graph and its topological order is the same as the order of D genes in the IGH locus. Thus, our analysis agrees with conclusion made by Briney et al., 2012 that the order of D genes forming tandem CDR3s follows their order in the IGH locus.

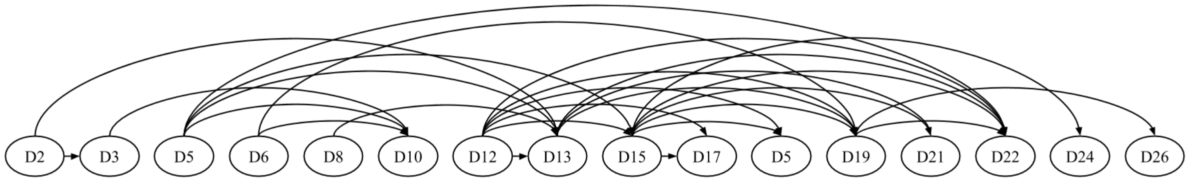

Figure A22. Graph on 16 vertices and 27 edges corresponding to abundant D-pairs. Each abundant D-pair is represented by an edge from its first D gene to its second D gene.

We compared usage of D genes in traceable CDR3s with usage of the first and second D genes in D-pairs. Six D genes with high usage (>5%) in traceable CDR3s (D3, D10, D13, D15, D19, and D22) also have high usage (>5%) in tandem CDR3s (Figure A23). However, eight abundant D genes in tandem CDR3s (D2, D5, D6, D8, D12, D17, D21, and D26) are not abundant in traceable CDR3s.

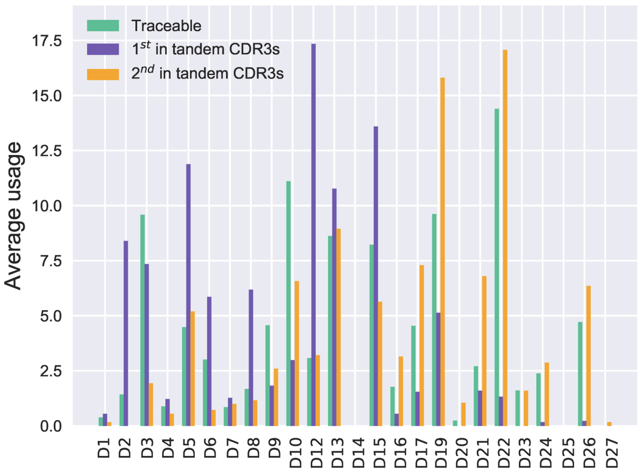

Figure A23. Usage of known D genes in traceable CDR3s (green) and tandem CDR3s as the 1st (purple) and 2nd (orange) D gene forming a tandem CDR3. The average usage was computed across usages in all HEALTHY datasets.

Table A14 presents the tandem bias and fraction of tandem CDR3s for all HEALTHY datasets. We classify a pair (*D*, *D'*) of D genes as *direct* (*reverse*) if all occurrences of *D* precede (follow) all occurrences of *D'* in the IGH locus. A pair (*D*, *D'*) is classified as *ambiguous* if it is neither direct, not reverse. Note that only pairs including D4 or D5 gene (that have two copies in the IGH locus) can be classified as ambiguous. We classify a tandem CDR3 as direct/reverse/ambiguous if it is formed by direct/reverse/ambiguous pair of D genes. The average percentages of direct, reverse and ambiguous CDR3s across all HEALTHY datasets are 82%, 6%, and 12%. On average, 88%, 91%, and 85% of sequences are productive in direct, reverse and ambiguous CDR3s across all HEALTHY datasets (**Figure A24**). Thus, the identified tandem CDR3s are likely to represent productive immunoglobulins rather than sample preparation artifacts.

dataset	tandem bias	% of tandem CDR3s	dataset	tandem bias	% of tandem CDR3s
Set 1	0.10	0.20	Set 8	0.10	0.18
Set 2	0.20	0.30	Set 9	0.04	0.25
Set 3	0.07	0.18	Set 10	0.06	0.16
Set 4	0.10	0.21	Set 11	0.16	0.14
Set 5	0.05	0.16	Set 12	0.09	0.13
Set 6	0.10	0.21	Set 13	0.06	0.11
Set 7	0.08	0.14	Set 14	0.09	0.16

Table A14. The tandem bias and the percentage of tandem CDR3s for all HEALTHY datasets. The column "% of tandem CDR3" shows the percentage of tandem CDR3 among all traceable CDR3s for each immunosequencing dataset.

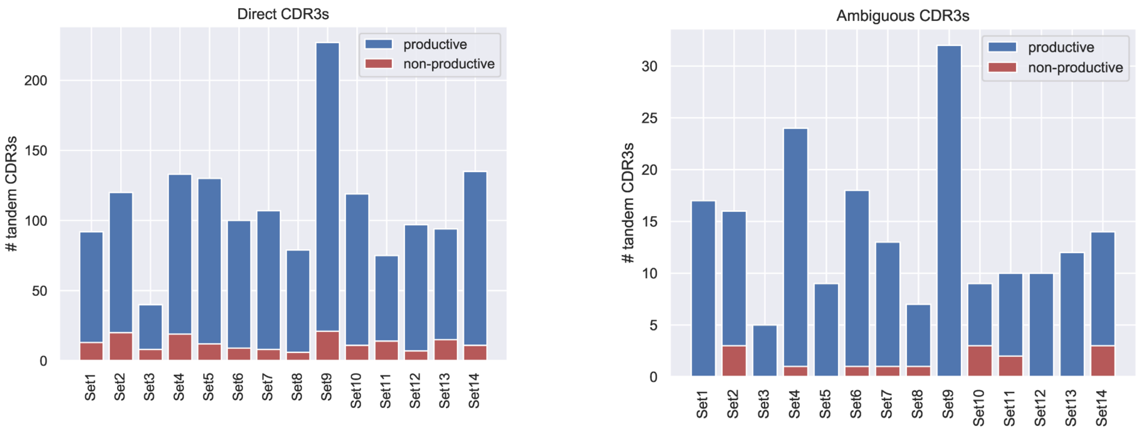

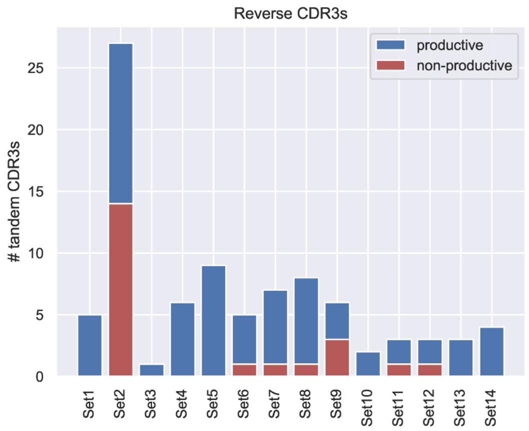

Figure A24. Productive and non-productive sequences in direct, ambiguous, and reverse CDR3s across 14 HEALTHY datasets.

Figure A25 demonstrates that the percentage and length of tandem CDR3s in the ALLERGY BM datasets is higher than in the ALLERGY PBMC and HIV PBMC datasets. Table A15 shows the numbers of sequences containing tandem CDR3s classified according to the immunoglobulin isotype in the ALLERGY and HIV datasets.

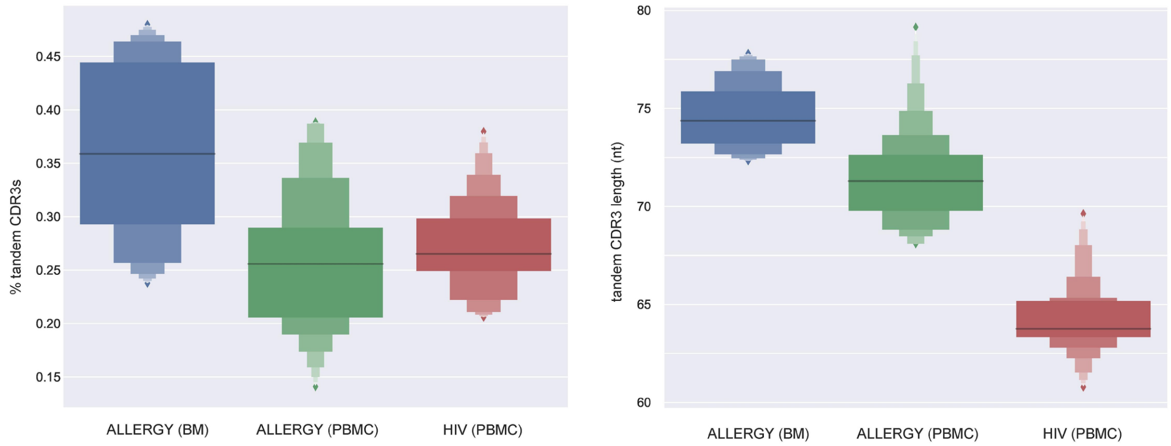

Figure A25. The percentages (left) and lengths (right) of tandem CDR3s in ALLERGY (bone marrow), ALLERGY (PBMC), and HIV (PBMC) datasets.

Dataset	IgM	IgG	IgE	IgA	Dataset	IgM	IgG	IgE	IgA
ALLERGY1	104	5	0	0	ALLERGY20	49	2	0	3
ALLERGY2	183	6	2	3	ALLERGY21	386	3	0	7
ALLERGY3	241	4	0	2	ALLERGY22	353	7	0	8
ALLERGY4	244	1	1	1	ALLERGY23	213	1	0	1
ALLERGY5	255	13	1	3	ALLERGY24	138	2	0	0
ALLERGY6	385	11	0	6	HIV1	12	29	0	0
ALLERGY7	194	5	0	8	HIV2	53	22	0	0
ALLERGY8	275	2	0	2	HIV3	8	32	0	0
ALLERGY9	49	4	0	12	HIV4	58	24	0	0
ALLERGY10	101	3	0	10	HIV5	44	17	0	0
ALLERGY11	147	6	0	9	HIV6	49	16	0	0

ALLERGY12	147	5	0	11	HIV7	11	26	0	0
ALLERGY13	175	7	0	5	HIV8	10	29	0	0
ALLERGY14	124	11	0	6	HIV9	18	39	0	0
ALLERGY15	130	7	0	4	HIV10	11	33	0	0
ALLERGY16	180	8	0	7	HIV11	8	27	0	0
ALLERGY17	142	3	0	6	HIV12	8	21	0	0
ALLERGY18	313	8	0	5	HIV13	5	20	0	0
ALLERGY19	57	3	1	3					

Table A15. The number of sequences containing tandem CDR3s classified according to the immunoglobulin isotype (ALLERGY and HIV datasets).

Figure A26 shows the tandem matrix constructed based on pairs of D genes forming tandem CDR3s in 15 datasets corresponding to the hepatitis patient 1776 analyzed in (Galson et al., 2015). The large number of entries in the D22 row in the lower part of this matrix suggests that the D22 gene is duplicated in this patient.

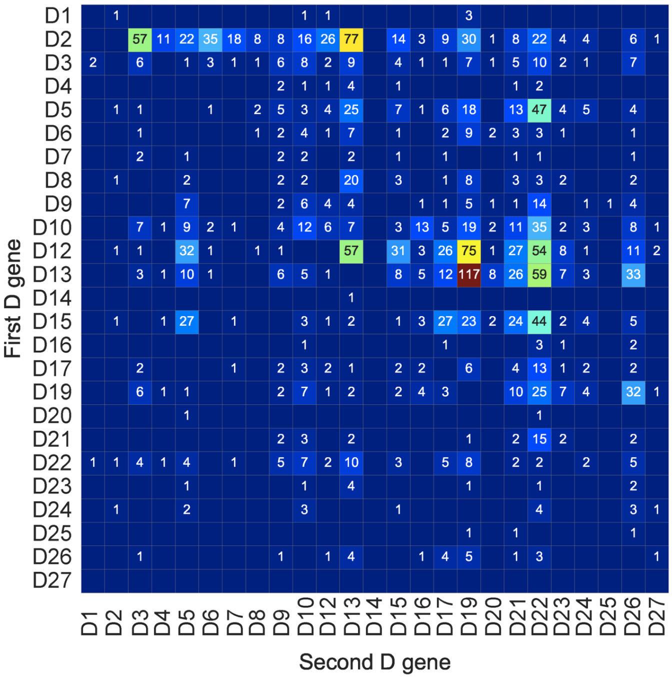

Figure A26. The tandem matrix for D genes forming tandem CDR3s in the datasets corresponding to the hepatitis patient 1776 (Galson et al., 2015).

Supplemental Note: Ultra-long CDR3s

We analyzed inter-D insertions in all 1900 tandem CDR3s across all HEALTHY datasets. These tandem CDR3s contain 1081 distinct inter-D insertions, varying in length from 0 to 153 nucleotides (Figure A27). 384 of them have length at least 10 nucleotides. Since most of them do not share significant similarities, they likely correspond to randomly generated sequences.

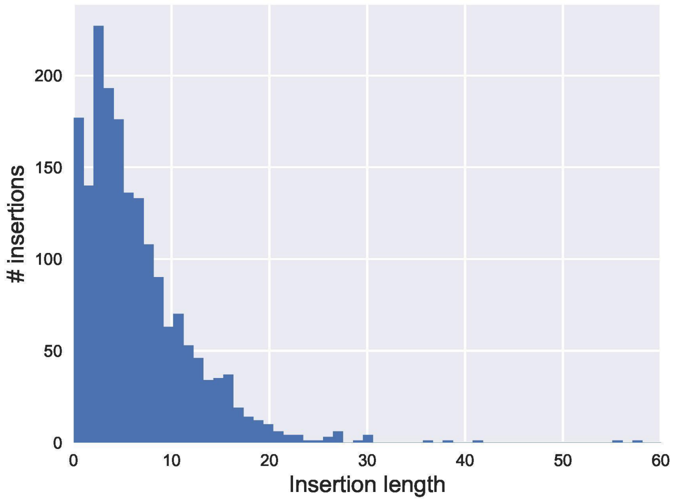

Figure A27. Distribution of lengths of inter-D insertions in tandem CDR3s across all HEALTHY datasets. Two ultra-long inter-D insertions (of length 153 nucleotides) are not shown.

Two longest inter-D insertions (denoted I_1 and I_2) appear in the Set 1 and have length 153 nucleotides. They are formed by genes D9 and D10, differ by a single nucleotide, and appear in CDR3s differing by six nucleotides (Figure A28, top). The inter-D insertion I_2 starts with the right RSS of D9 and ends with the left RSS of D10 (Figure A28, bottom).

Additionally, we detected RSS skipping in 13 tandem CDR3s from the ALLERGY datasets. We have also detected 69 ultra-long CDR3s (9 of them are productive) containing a single D gene and some genomic fragment from IGH locus. The origin of these 69 additional CDR3s remains unclear, we suggest that they result from partially off-target recombination involving the CAC motif (Hu et al., 2015; Zhao et al., 2016; Jain et al., 2018). For example, all 9 productive CDR3s are formed skipping of the right RSS of D22. Instead of it, somatic recombination uses a cryptic RSS (CACAGCA + ACCCAAACA) located at the distance 129 nt from the end of D22 (Figure A29). As a result, the found CDR3s contain both a fragment of D22, the right RSS of D22, and a fragment of IGH locus following it. We also have not found a strong association between found ultra-long CDR3s and specific V or J genes.

Recent studies demonstrated importance of such genomic insertions, e.g., in the case of the LAIR insertion in malaria specific antibodies (Tan et al., 2016). We found 9 productive CDR3s among 83 detected ultra-long CDR3s (the example mentioned in the text is not productive) suggesting that the RSS skipping mechanism may contribute to the diversity of antibody repertoire.

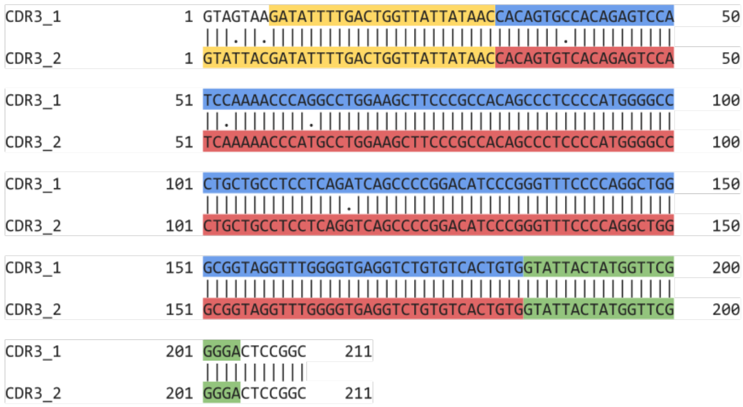

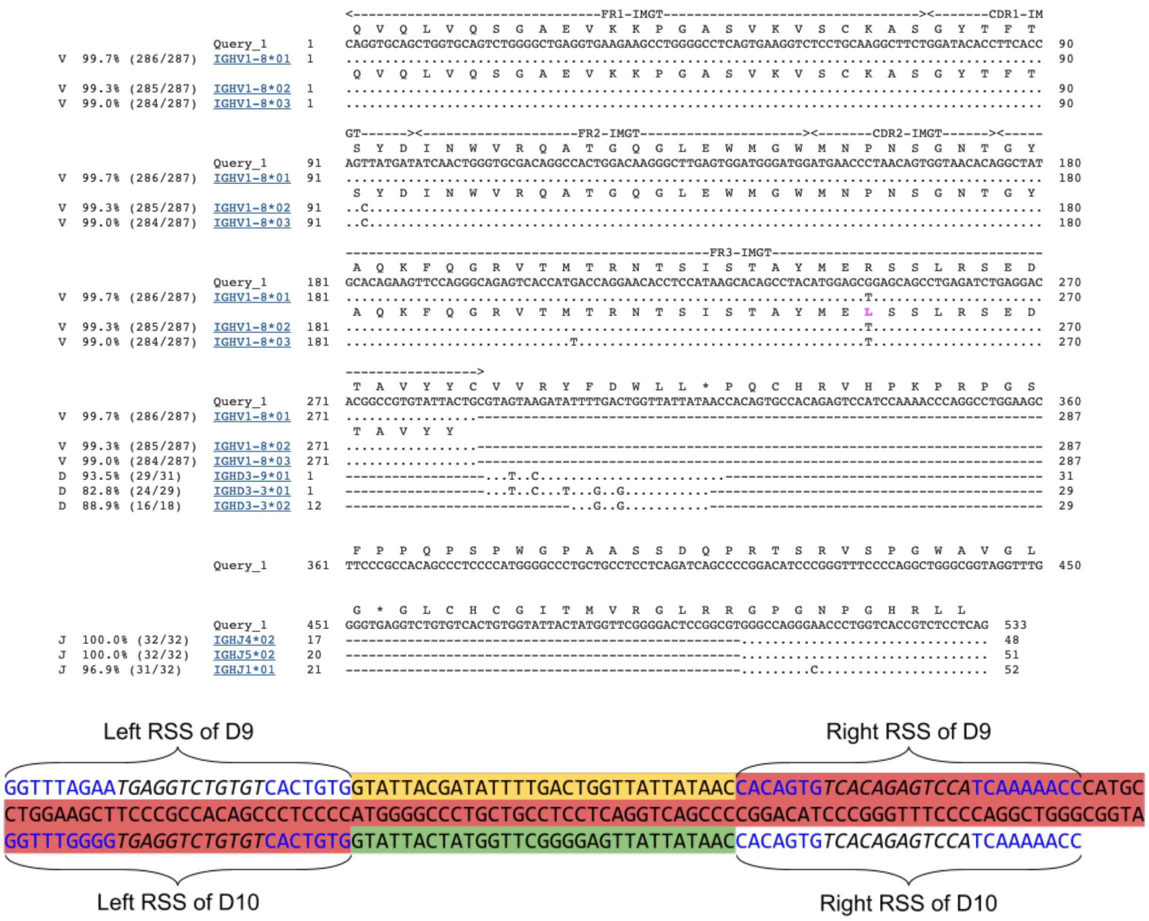

Figure A28. RSS skipping in ultra-long tandem CDR3s. (Top) Alignment of tandem CDR3s formed by genes D9 (yellow) and D10 (green) and containing ultra-long inter-D insertions I_1 (blue) and I_2 (red). (Middle) Alignment of the full-length sequence corresponding to the first ultra-long CDR3 against germline V, D, and J genes generated using IgBlast. Note that the illustrated sequence is not productive. (Bottom) Fragment of the IGH locus starting with the left RSS sequence of the D9 gene and ending with the right RSS sequence of the D10 gene. Left RSS sequences are shown as concatenates of a nonamer (shown in blue), a 12-nucleotide long spacer (shown in italic), and a heptamer (shown in blue). Right RSS sequences are shown as concatenates of a heptamer (shown in blue), a 12-nucleotide long spacer (shown in italic), and a nonamer (shown in blue).

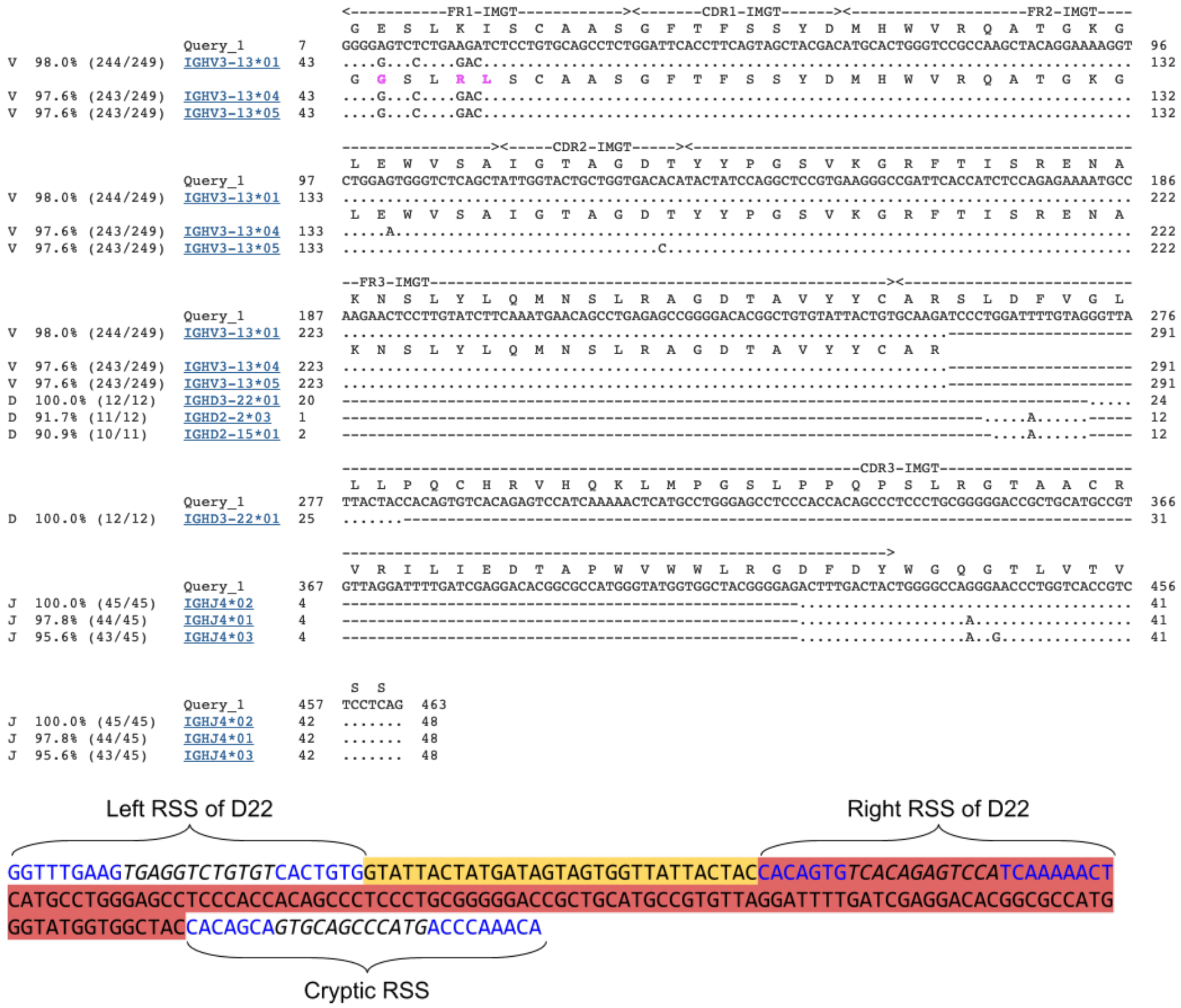

Figure A29. RSS skipping and off-target recombination results in a productive ultra-long CDR3 (found in ALLERGY13 dataset). (Top) Alignment of the full-length sequence corresponding to the first ultra-long CDR3 against germline V, D, and J genes generated using IgBlast. (Bottom) Fragment of the IGH locus starting with the left RSS sequence of the D22 gene and ending with a cryptic RSS. The left RSS of D22 is shown as concatenates of a nonamer (shown in blue), a 12-nucleotide long spacer (shown in italic), and a heptamer (shown in blue). The right RSS of D22 and the cryptic RSS are shown as concatenates of a heptamer (shown in blue), a 12-nucleotide long spacer (shown in italic), and a nonamer (shown in blue).

Supplemental Note: *De novo* reconstruction of human J genes

All human J genes are located in a 2 kb long region in the human IGH locus (Table A16). Table A17 and Figure A30 show allelic variants of human J genes listed in the IMGT database.

Name	IMGT name	Position (bp)	Length (nt)
J1	IGHJ1	105,865,405	52
J2	IGHJ2	105,865,197	53
J3	IGHJ3	105,864,585	50
J4	IGHJ4	105,864,213	48
J5	IGHJ5	105,863,812	51
J6	IGHJ6	105,863,196	63

Table A16. Positions and lengths of J genes on the 14th chromosome in the human genome. Since the IGH locus starts at the end of the 14th chromosome, positions are given with respect to its complementary sequence (assembly GRCh38.p12).

J gene	IMGT allele	ID

J1	IGHJ1*01	J1
J2	IGHJ2*01	J2
J3	IGHJ3*01	J3*
	IGHJ3*02	J3
J4	IGHJ4*01	J4*
	IGHJ4*02	J4
	IGHJ4*03	J4**
J5	IGHJ5*01	J5
	IGHJ5*02	J5*
J6	IGHJ6*01	J6*
	IGHJ6*02	J6
	IGHJ6*03	J6**
	IGHJ6*04	J6***

Table A17. Information about variants of J genes and their correspondence with alleles listed in the IMGT database.

```
J3     TGATGCTTTTGATATCTGGGGCCAAGGGACAATGGTCACCGTCTCTTCAG
J3*    TGATGCTTTTGATGTCTGGGGCCAAGGGACAATGGTCACCGTCTCTTCAG

J4     ACTACTTTGACTACTGGGGCCAGGGAACCCTGGTCACCGTCTCCTCAG
J4*    ACTACTTTGACTACTGGGGCCAAGGAACCCTGGTCACCGTCTCCTCAG
J4**   GCTACTTTGACTACTGGGGCCAAGGGACCCTGGTCACCGTCTCCTCAG

J5     ACAACTGGTTCGACTCCTGGGGCCAAGGAACCCTGGTCACCGTCTCCTCAG
J5*    ACAACTGGTTCGACCCCTGGGGCCAGGGAACCCTGGTCACCGTCTCCTCAG

J6     ATTACTACTACTACTACGGTATGGACGTCTGGGGCCAAGGGACCACGGTCACCGTCTCCTCAG
J6*    ATTACTACTACTACTACGGTATGGACGTCTGGGGGCAAGGGACCACGGTCACCGTCTCCTCAG
J6**   ATTACTACTACTACTACTACATGGACGTCTGGGGCAAAGGGACCACGGTCACCGTCTCCTCAG
J6***  ATTACTACTACTACTACGGTATGGACGTCTGGGGCAAAGGGACCACGGTCACCGTCTCCTCAG
```

Figure A30. Allelic variants of human J genes. Differences between various variants are highlighted in red.

De novo reconstruction of J genes requires immunosequencing reads that cover the entire J genes. This is not the case for many immunosequencing datasets, including all HEALTHY datasets. We benchmarked how IgScout reconstructs human J genes using the ALLERGY (rather than HEALTHY) datasets since reads in these datasets cover the entire J segment.

We combined four datasets corresponding to the first allergic donor and identified 5,940,059 fragments of J genes in reads from this dataset using IgReC, resulting in the set of strings $J_{trimmed}$. Afterwards, we applied IgScout to infer J genes from these strings. Since J genes are longer than D genes, we increased the parameter *k-mer size* from 15 to 40 (all 40-mers in J genes are unique, i.e., they appear in a single J gene). The human J genes (from J1 to J6) contain 83 40-mers (192 40-mers including their alleles listed in the IMGT database). The $J_{trimmed}$ dataset contains all 40-mers appearing in six human J genes.

We classify a k-mer as *known* if it occurs in a human J gene (from J1 to J6), *mutated* if it differs from a known k-mer by a single nucleotide, and *trimmed* if it contains a known (k-2)-mer. All other k-mers are classified as *foreign.* 41% of strings in the $J_{trimmed}$ dataset contain a known 40-mer. 43% strings in the $J_{trimmed}$ dataset contain either a known, or a mutated, or a trimmed 40-mer.

Since the number of J genes is smaller than the number of D genes (6 vs 25), we increased the *fraction* parameter to 0.02 for the case of the J gene finding, i.e., a k-mer is classified as *common* if its abundance exceeds 2% of the number of sequences in the $J_{trimmed}$ set. Figure A31 presents distribution of abundances of all 47 common 40-mers in the $J_{trimmed}$ set. Figure A32 shows that the usages of human J genes are similar for various ALLERGY datasets.

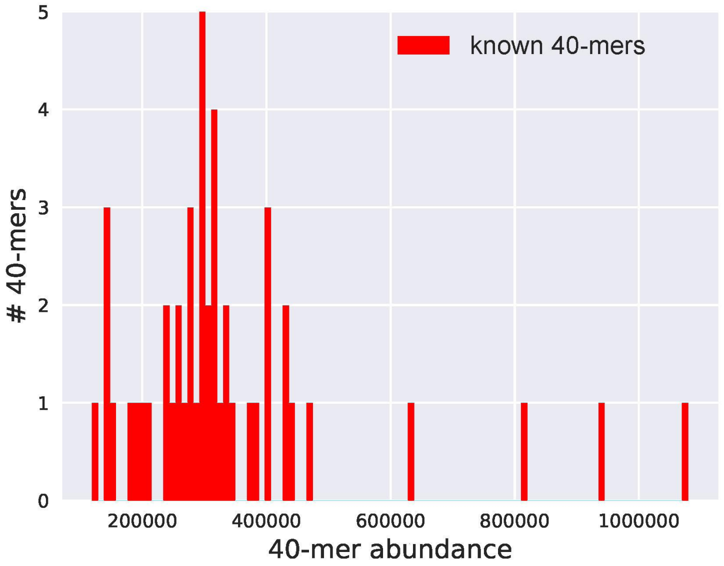

Figure A31. Abundances of all 47 common 40-mers in the $J_{trimmed}$ set. 47 common 40-mers in the $J_{trimmed}$ set have abundances varying from 119,082 to 1,079,233. The *y*–axis represents the number of common 40-mers with given abundance. All 47 common 40-mers are known (shown as red bars). Each bar represents the number of common 40-mers with given abundance. The histogram represents 29 bins of width 10,000 each.

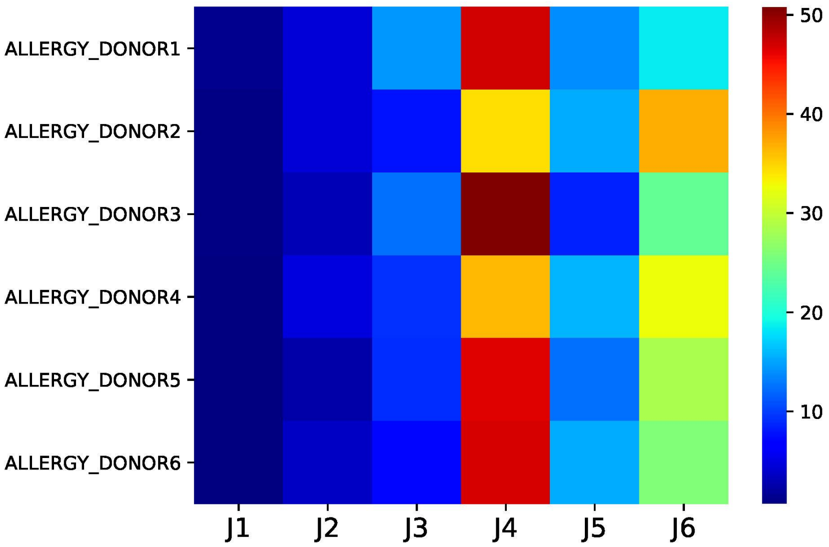

Figure A32. Usage of human J genes in the ALLERGY datasets. We merged four datasets corresponding to each of six ALLERGY donors and computed the J gene usage for each donor.

We applied IgScout to the $J_{trimmed}$ dataset with k = 40. Ranks and abundances of known 40-mers are shown in Figure A33. IgScout reconstructed four strings representing the complete sequences of the J3, J4, J5, and J6 genes (Figure A34). The J1 and J2 genes were not reconstructed by IgScout since their most abundant 40-mers do not pass the *fraction* threshold (the most abundant 40-mer from the J1 and J2 genes are supported by 30,000 and 99,000 CDR3s, respectively.

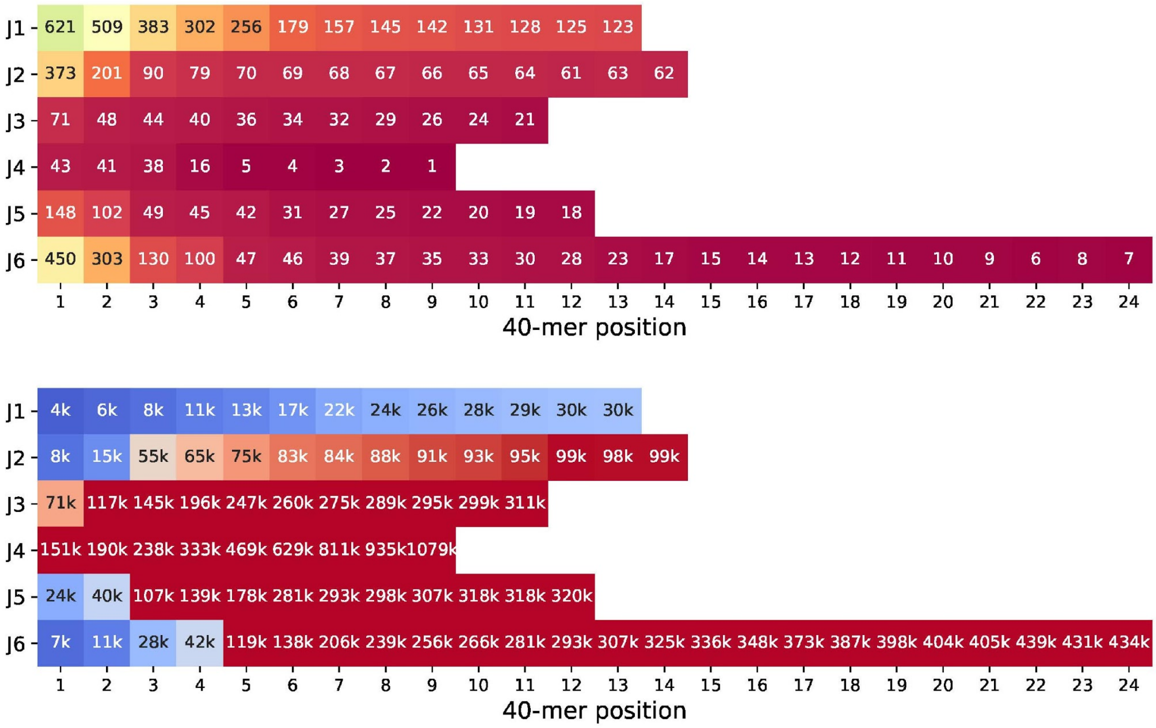

Figure A33. Ranks (top) and abundances (bottom) of 40-mers from human J genes ($J_{trimmed}$ set). Details of this visualization are described in the legend for Figure A3. Abundances exceeding 100,000 are shown in red.

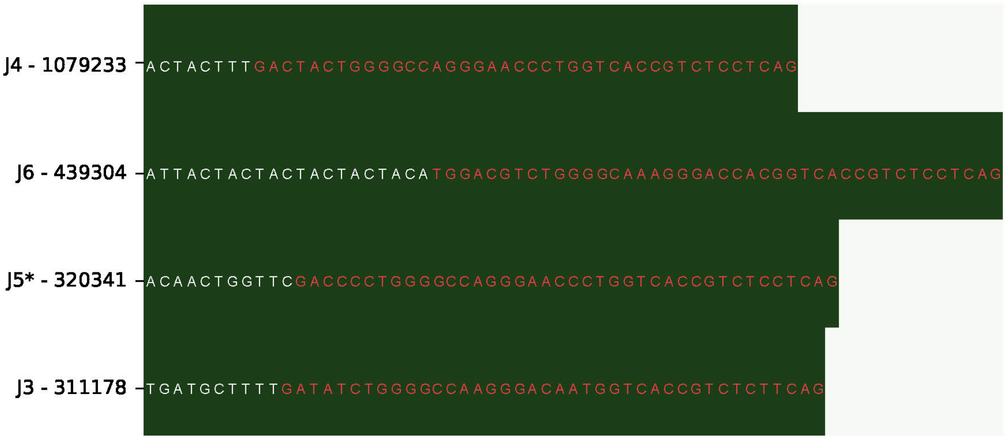

Figure A34. Results of the IgScout algorithm on the $J_{trimmed}$ dataset. Details of this visualization are described in the legend for Figure 2.

Since IgDiscover (Corcoran et al., 2016) is limited to *de novo* reconstruction of V genes, IMPre (Zhang et al., 2016) is the only available tool for *de novo* reconstruction of J genes. Since IMpre demonstrates the best results on sequences trimmed by the ends of J genes, we trimmed suffixes of reads corresponding to constant regions. We applied IgScout and IMPre to 6 donors from the ALLERGY dataset and compared the J genes inferred by IgScout and IMPre (Figure A35).

We classify an inferred segment as *erroneous* if it was formed by an addition of incorrect nucleotides to the start of a J gene. IgScout reconstructed four out of six human J genes over their entire lengths (including the known variant J6**) and made no errors. IMPre reconstructed all six J genes (including the known variants J3* and J6**) but made seven errors. While IgScout reconstructs complete D genes, IMPre misses 2.3 nucleotides on average at the start of the reconstructed J genes.

Both IgScout and IMPre reported 3 novel variants of J genes each. However, since all these six variants are different, they are likely caused by frequent SHMs in J genes and thus require additional tuning of both tools for *de novo* reconstruction of J genes.

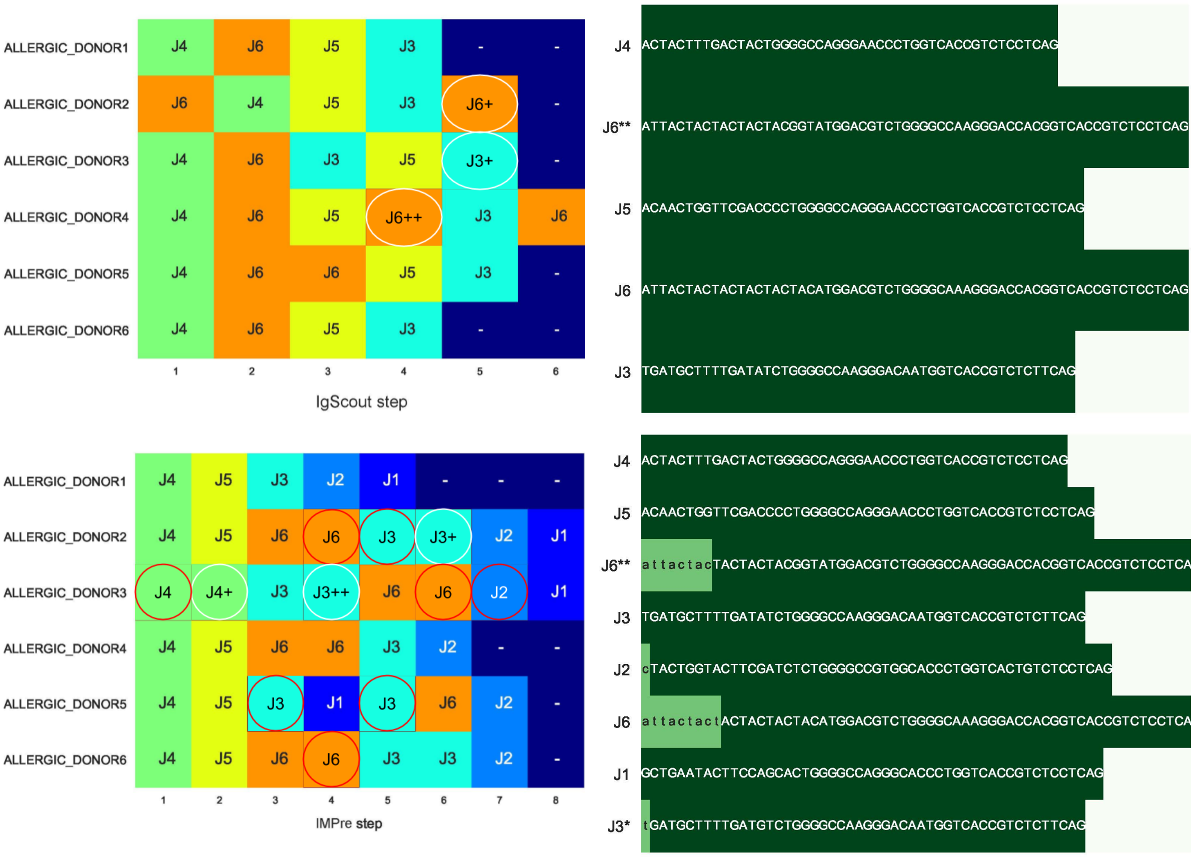

Figure A35. ***De novo*** **reconstructions of J genes for six ALLERGY patients using using IgScout (top) and IMPre (bottom).** Details of this visualization are described in the legends for Figure 3 and Figure A3. Some reported sequences represent inaccurately reconstructed J genes (e.g., a J gene with several added nucleotides at the start) that we represented using red circles.

Supplemental Note: List of tandem CDR3s

Figure A36 lists all 114 tandem CDR3s in the Set 1 dataset.

D12	gtggaTATAGTGGCTACGATtac
D5	gtGGATACAGCTATGGTTAC
CDR3	GCGAGAGAGGGGGGCGGGTATAGTGGCTACGATACGCGGATACAGCTATGGTTACGAGGGTACTACTACTACGGTATGGACGTC
D12	gtggatATAGTGGCTACGATTac
D5	gtggATACAGCTATGGTTAC
CDR3	GCGAGATGCGGGATGGATAGTGGCTACGATTGGGCATACAGCTATGGTTACGGGCCTCGGTACTACTACTACGGTATGGACGTC
D3	gtattacGATTTTTGGAGTGGTTATtatacc
D7	ggtaTAACTGGAACTAC
CDR3	GCGAGCCTAAGATTTTTGGAGTGGTTATCACTAACTGGAACTACTACCTACTACTACTACGGTATGGACGTC
D2	aggatattgtagTAGTACCAGCTGCTATAcc
D8	AGGATATTGTACTAAtggtgtatgctatacc
CDR3	GCGAGAGAAGTTGGACCCTAGTACCAGCTGCTATAGTCTCTAGGATATTGTACTAATGGCTACTAGACTAC
D19	gggtaTAGCAGTGGCTGgtac
D23	tGACTACGGTGGtaactcc
CDR3	GCGAGAGTAATAGCAGTGGCTGCTACCGACTACGGTGGGCTCGATGCTTTTGATATC
D2	aggatATTGTAGTAGTACCAGCTGCTATAcc
D12	GTGGATATAGTGGCTACGATtac
CDR3	GCGAGAGAGCGGGGACCACGGGTAGATTGTAGTAGTACCAGCTGCTATAGTGGATATAGTGGCTACGATTACTTTGACTAC
D10	gtattactaTGGTTCGGGGAGTTATtataac
D22	gtattaCTATGATAGTAGTGgttattactac
CDR3	GCGAGAGATTTTCAGGTGGTTCGGGGAGTTATACGGGCCCCGTCTATGATAGTAGTGCCACAAGGGGTTGACTAC
D13	gggtaTAGCAGCAGCTGGTAc
D20	gGTATAACTGGAACGAC
CDR3	GCGAGACTGGTAGCAGCAGCTGGTAGTATAACTGGAACGACGGGCTTTGACTAC
D21	agcatATTGTGGTGGTGACTGCtattcc
D16	gtattatgattacgtttgGGGGAGTTATCgttatacc
CDR3	GCGAGATTAAGATCGGTCCATTGTGGTGGTGACTGCCCCCCTAGGGGAGTTATCCCCGAGGATGACTAC
D5	gtgGATACAGCTATGGTtac
D15	AGGATATTGTAGTGGTGGTAGCTGCTACtcc
CDR3	GCGAGACGTGGATACAGCTATGGTCCAGGATATTGTAGTGGTGGTAGCTGCTACGATACCTCTGGGGTGCCATACTACTACGGTATGGACGTC

Figure A36. List of 114 tandem CDR3s in the Set 1 dataset. All 114 tandem CDR3s can be found here.

Supplemental references

Briney B, Inderbitzin A, Joyce C, Burton DR. Commonality despite exceptional diversity in the baseline human antibody repertoire. Nature. 2019 Feb;566(7744):393-397.

Elhanati Y, Sethna Z, Marcou Q, Callan CG Jr, Mora T, Walczak AM. Inferring processes underlying B-cell repertoire diversity. Philos Trans R Soc Lond B Biol Sci. 2015;370:1676.

Ellebedy AH, Jackson KJ, Kissick HT, Nakaya HI, Davis CW, Roskin KM, McElroy AK, Oshansky CM, Elbein R, Thomas S, Lyon GM, Spiropoulou CF, Mehta AK, Thomas PG, Boyd SD, Ahmed R. Defining antigen-specific plasmablast and memory B cell subsets in blood following viral infection and vaccination of humans. Nature Immunol. 2016;17(10):1226-34.

Friedensohn S, Lindner JM, Cornacchione V, Iazeolla M, Miho E, Zingg A, Meng S, Traggiai E, Reddy ST. Synthetic Standards Combined With Error and Bias Correction Improve the Accuracy and Quantitative Resolution of Antibody Repertoire Sequencing in Human Naïve and Memory B Cells. Front Immunol. 2018 Jun 20;9:1401.

Galson JD, Trück J, Fowler A, Clutterbuck EA, Münz M, Cerundolo V, Reinhard C, van der Most R, Pollard AJ, Lunter G, Kelly DF. Analysis of B Cell Repertoire Dynamics Following Hepatitis B Vaccination in Humans, and Enrichment of Vaccine-specific Antibody Sequences. EBioMedicine. 2015 Nov 24;2(12):2070-9.

Gupta NT, Adams KD, Briggs AW, Timberlake SC, Vigneault F, Kleinstein SH. Hierarchical Clustering Can Identify B Cell Clones with High Confidence in Ig Repertoire Sequencing Data. J Immunol. 2017 Mar 15;198(6):2489-2499.

Hu J, Zhang Y, Zhao L, Frock RL, Du Z, Meyers RM, Meng FL, Schatz DG, Alt FW. Chromosomal Loop Domains Direct the Recombination of Antigen Receptor Genes. Cell. 2015; 163(4):947-59.

Jain S, Ba Z, Zhang Y, Dai HQ, Alt FW. CTCF-Binding Elements Mediate Accessibility of RAG Substrates During Chromatin Scanning. Cell. 2018; 174(1):102-116.e14.

Magri G, Comerma L, Pybus M, Sintes J, Lligé D, Segura-Garzón D, Bascones S, Yeste A, Grasset EK, Gutzeit C, Uzzan M, Ramanujam M, van Zelm MC, Albero-González R, Vazquez I, Iglesias M, Serrano S, Márquez L, Mercade E, Mehandru S, Cerutti A. Human Secretory IgM Emerges from Plasma Cells Clonally Related to Gut Memory B Cells and Targets Highly Diverse Commensals. Immunity. 2017 Jul 18;47(1):118-134.e8.

Soto C, Bombardi RG, Branchizio A, Kose N, Matta P, Sevy AM, Sinkovits RS, Gilchuk P, Finn JA, Crowe JE Jr. High frequency of shared clonotypes in human B cell receptor repertoires. Nature. 2019 Feb;566(7744):398-402.

Souto-Carneiro MM, Sims GP, Girschik H, Lee J, Lipsky PE. Developmental changes in the human heavy chain CDR3. J Immunol. 2005;175(11):7425-36.

Stern JN, Yaari G, Vander Heiden JA, Church G, Donahue WF, Hintzen RQ, Huttner AJ, Laman JD, Nagra RM, Nylander A, Pitt D, Ramanan S, Siddiqui BA, Vigneault F, Kleinstein SH, Hafler DA, O'Connor KC. B cells populating the multiple sclerosis brain mature in the draining cervical lymph nodes. Sci Transl Med. 2014 Aug 6;6(248):248ra107.

Tan J, Pieper K, Piccoli L, Abdi A, Perez MF, Geiger R, Tully CM, Jarrossay D, Maina Ndungu F, Wambua J, Bejon P, Fregni CS, Fernandez-Rodriguez B, Barbieri S, Bianchi S, Marsh K, Thathy V, Corti D, Sallusto F, Bull P, Lanzavecchia A. A LAIR1 insertion generates broadly reactive antibodies against malaria variant antigens. Nature. 2016; 529(7584):105-109.

Zhao L, Frock RL, Du Z, Hu J, Chen L, Krangel MS, Alt FW. Orientation-specific RAG activity in chromosomal loop domains contributes to Tcrd V(D)J recombination during T cell development. J Exp Med. 2016; 213(9):1921-36.

Waltari E, Jia M, Jiang CS, Lu H, Huang J, Fernandez C, Finzi A, Kaufmann DE, Markowitz M, Tsuji M, Wu X. 5' Rapid Amplification of cDNA Ends and Illumina MiSeq Reveals B Cell Receptor Features in Healthy Adults, Adults With Chronic HIV-1 Infection, Cord Blood, and Humanized Mice. Front Immunol. 2018 Mar 26;9:628.

Ye J, Ma N, Madden TL, Ostell JM. IgBLAST: an immunoglobulin variable domain sequence analysis tool. Nucleic Acids Res. 2013;41:W34-40.
